# Human liver microbiota modeling strategy at the early onset of fibrosis

**DOI:** 10.1186/s12866-023-02774-4

**Published:** 2023-01-30

**Authors:** Camille Champion, Radu M. Neagoe, Maria Effernberger, Daniela T. Sala, Florence Servant, Jeffrey E. Christensen, Maria Arnoriaga-Rodriguez, Jacques Amar, Benjamin Lelouvier, Pascale Loubieres, Vincent Azalbert, Matthieu Minty, Charlotte Thomas, Vincent Blasco-Baque, Fabrice Gamboa, Herbert Tilg, Marina Cardellini, Massimo Federici, Jose-Manuel Fernández-Real, Jean Michel Loubes, Rémy Burcelin

**Affiliations:** 1 Institut National de La Santé Et de La Recherche Médicale (INSERM), Toulouse, France; 2 Unité Mixte de Recherche (UMR) 1297, Institut Des Maladies Métaboliques Et Cardiovasculaires (I2MC), Team 2: ‘Intestinal Risk FactorsDiabetesDyslipidemia’, Université Paul Sabatier (UPS), F-31432 Toulouse Cedex 4, France; 3 Institut de Mathématiques de Toulouse, Université Paul Sabatier, Toulouse, France; 4 Second Department of Surgery, Emergency Mureş County Hospital, University of Medicine Pharmacy, Science and Technology “George Emil Palade” Tîrgu Mures, Târgu Mureș, Romania; 5 Department of Internal Medicine I, Gastroenterology, Hepatology, Endocrinology & Metabolism, Medical University of Innsbruck, Innsbruck, Austria; 6 VAIOMER, 516 Rue Pierre Et Marie Curie, 31670 Labège, France; 7 Department of Diabetes, Endocrinology and Nutrition, University Hospital of Girona ‘Dr Josep Trueta’, Girona, Spain; 8 Institut d’Investigacio Biomedica de Girona IdibGi, Girona, Spain; 9 CIBER Fisiopatologia de La Obesidad Y Nutricion, Girona, Spain; 10 Therapeutics Department, Rangueil Hospital, Toulouse, France; 11 Department of Systems Medicine, University of Rome “Tor Vergata”, Via Montpellier 1, 00133 Rome, Italy

**Keywords:** Biomathematics, Liver diseases, Metabolic disease, Microbiota, Tissue microbiota

## Abstract

**Background:**

Gut microbiota is involved in the development of liver diseases such as fibrosis. We and others identified that selected sets of gut bacterial DNA and bacteria translocate to tissues, notably the liver, to establish a non-infectious tissue microbiota composed of microbial DNA and a low frequency live bacteria. However, the precise set of bacterial DNA, and thereby the corresponding taxa associated with the early stages of fibrosis need to be identified. Furthermore, to overcome the impact of different group size and patient origins we adapted innovative statistical approaches. Liver samples with low liver fibrosis scores (F0, F1, F2), to study the early stages of the disease, were collected from Romania(*n* = 36), Austria(*n* = 10), Italy(*n* = 19), and Spain(*n* = 17). The 16S rRNA gene was sequenced. We considered the frequency, sparsity, unbalanced sample size between cohorts to identify taxonomic profiles and statistical differences.

**Results:**

Multivariate analyses, including adapted spectral clustering with L1-penalty fair-discriminant strategies, and predicted metagenomics were used to identify that 50% of liver taxa associated with the early stage fibrosis were Enterobacteriaceae, Pseudomonadaceae, Xanthobacteriaceae and Burkholderiaceae. The Flavobacteriaceae and Xanthobacteriaceae discriminated between F0 and F1. Predicted metagenomics analysis identified that the preQ0 biosynthesis and the potential pathways involving glucoryranose and glycogen degradation were negatively associated with liver fibrosis F1-F2 vs F0.

**Conclusions:**

Without demonstrating causality, our results suggest first a role of bacterial translocation to the liver in the progression of fibrosis, notably at the earliest stages. Second, our statistical approach can identify microbial signatures and overcome issues regarding sample size differences, the impact of environment, and sets of analyses.

**Trial registration:**

TirguMECCH ROLIVER Prospective Cohort for the Identification of Liver Microbiota, registration 4065/2014. Registered 01 01 2014.

**Supplementary Information:**

The online version contains supplementary material available at 10.1186/s12866-023-02774-4.

## Introduction

Non-alcoholic fatty liver disease (NAFLD) is a common consequence of obesity and type 2 diabetes [1, 2]. In NAFLD, the origin of inflammation and hepatocyte injury is related to dietary lipids, bile acids, adipokines and cytokines, to cite a few. Furthermore, gut microbiota seems to be one of the key players of NAFLD development [3, 4]. Markers and receptors of microbiota-related injury features have been described in this disorder such as TLRs, NLRs, and NLRP3 [5–8] as well as the activation of the innate and adaptive immune systems [9]. In early sets of experiments, we initially showed that hepatic steatosis in the obese diabetic mouse was due to an increased circulating concentration of lipopolysaccharides (LPS) i.e. metabolic endotoxemia [10]. Lipoproteins transport LPS [11] to tissues, triggering the CD14/TRL4 pathway that increases liver inflammation and fat deposition [10]. Gut bacteria were also reported to translocate through the intestinal tract to tissues [12], such as the adipose depots and the liver, establishing a tissue microbiota as observed in rodents [13–15] and humans [16–18]. This tissue microbiota could trigger liver inflammation and the onset of fibrosis [13]. This mechanism activates immune cells, including Kupffer cells, to release various pro-inflammatory cytokines and chemokines [19] damaging the surrounding tissues initiating hence, fibrosis. This hypothesis is now largely supported by recent major advances in NAFLD research, which show gut and blood microbiota dysbiosis of patients with advanced stages of NAFLD [20–22]. Hence, the identification of specific groups of translocated bacteria from the dysbiotic gut microbiota could aid in the design of novel therapeutic strategies. It is noteworthy that in other instances, such as in cancer authors did identify, isolate, and showed that intracellular bacteria control the efficacy of anti-cancer drugs [23–28]. Hence, this key observation reinforces our long term goal which is to show that tissue microbiota either through the bacterial DNA or the live bacteria could initiate metabolic diseases.

To bring more light to this goal, we have sequenced and identified the bacterial 16S rRNA gene from liver biopsies of a cohort of 36 Romanian, 17 Spanish, 19 Italians and 10 Austrian patients with early stages liver fibrosis. It is noteworthy that the discrimination between patients with F0 and F1 scores could depend upon the biopsy sample or the practitioner. We here challenge this point by using an agnostic approach where only the liver bacterial DNA sequences would be classifiers of the F0 and F1 scores. We then, highlighted that such approach was indeed a good classifier of the patients. Eventually, we could design hypotheses regarding the putative causal role of liver microbiota at the onset of liver fibrosis. We used this database to evaluate the efficacy of Principal Coordinate Analysis (PCoA) to visualize the different liver fibrosis group scores using Wilcoxon-Mann–Whitney statistical tests [29]. Eventually, since the overall database of patients is issued from different cohorts we anticipated some degree of heterogeneity within the overall cohort. Therefore, we adapted and developed a specific statistical approach i.e. L1 spectral clustering with fairness. Without demonstrating causality, this approach establishes inter-relations between liver 16SrRNA bacterial DNA and low scores of liver fibrosis. Although, we did not demonstrate, in the present study, the existence of live bacteria our data are hence, strongly suggestive that some translocated bacteria could putatively be causal to the early onset of the disease. Thanks to our original mathematical approach, we could demonstrate that our results are adapted to the group size, the patient origins and sequencing batches. Overall, we drew a first partial “European microbial profile” of patients at early stages of liver fibrosis.

## Materials and methods

### Subjects

A multicentric observational study was conducted in the Second Department of Surgery, Emergency Mureş County Hospital of Romania, the Department of Systems Medicine of the Tor Vergata University of Rome, the Institut d’Investigacio Biomedica de Girona IdibGi, the Endocrinology and Nutrition Department of Dr. Josep Trueta University Hospital, and the University Hospital of Innsbruck. Exclusion criteria were serious liver diseases (eg hemochromatosis, alcoholic fatty liver disease, Hepatitis B and Hepatitis C infection, chronic diseases, inflammatory systemic diseases, acute or chronic infections in the previous month, use of antibiotic, antifungal, antiviral drugs, proton-pump inhibitors, anti-obesity drugs, laxatives, excessive use of vitamin D supplementation, fiber supplements or probiotics or participation in a weight loss program or weight change of 3 kg during the previous 6 weeks, pregnancy or breastfeeding, or major psychiatric antecedents; neurological diseases, history of trauma or injured brain, language disorders, and excessive alcohol intake (≥ 40 g/day in women or 80 g OH/day in men) or intravenous drug abuse, and previous bariatric surgery.

The cohort consists of 82 Caucasian patients where 34 were diagnosed with fibrosis stage 0 (F0); 37 stage 1 (F1) and 11 stage 2 (F2), as diagnosed from histological analyses of liver biopsies (Table 1). The patients suffered from morbid obesity with a mean BMI 42.6 (± 7.3). The mean waist circumference was 121.49 (± 18.73) in male and 123.23 (± 18.26) in female participants.

**Table 1:** Baseline characteristics of patients with biopsy-proven fibrosis

Characteristics	All patients *N* = 82	Stage F0 *N* = 34	Stage F1 *N* = 37	Stage F2 *N* = 11	*p* Value F0vsF1	*p* Value F2vsF0	*p* Value F2vsF1
Age (years)	41.50 ± 11.52	39.5 ± 12.77	39 ± 9.53	50 ± 9.15	0.99	0.16	0.03*
Female (n)	47 (57%)	15 (18%)	26 (32%)	6 (7.3%)	0.65	0.99	0.97
Height (m)	1.67 ± 0.08	1.67 ± 0.08	1.7 ± 0.08	1.62 ± 0.07	0.99	0.61	0.14
Smoker (n)	22 (27%)	10 (12%)	10 (12%)	2 (3%)	0.99	0.99	0.97
Weight (kg)	118.5 ± 23.99	120 ± 22.55	118 ± 21.59	115.8 ± 35.77	0.99	0.99	0.97
BMI (kg/m^2^)	42.65 ± 7.73	43.25 ± 6.9	41.6 ± 7.2	41.52 ± 11.41	0.99	0.99	0.97
Waist (cm)	121 ± 18.37	124.5 ± 19.4	120 ± 15.61	120 ± 24.12	0.99	0.99	0.99
Blood Glucose (mg/dl)	95.7 ± 25.76	95 ± 27.46	99 ± 21.22	95 ± 34.63	0.99	0.99	0.97
Treated Diabetes (n)	7 (8.5%)	1 (1.2%)	2 (2%)	4 (4.7%)	0.99	0.02 *	0.027 *
Systolic (mm Hg)	130 ± 19.47	130.5 ± 20.76	124 ± 17.43	134 ± 18.45	0.65	0.99	0.73
Diastolic (mm Hg)	80.0 ± 11.59	80.5 ± 11.4	75 ± 10.31	90 ± 15.3	0.88	0.99	0.97
Treated Hypertension (n)	20 (24%)	8 (9.7%)	5 (6%)	7 (8.2%)	0.99	0.15	0.027 *
Treated Dyslipidemia (n)	6 (7.3%)	2 (2%)	3 (3.6%)	1 (1.2%)	0.99	0.99	0.99
Total Cholesterol (mg/dL)	189.1 ± 39.78	190.0 ± 36.93	200.0 ± 43.11	167.0 ± 38.71	0.99	0.99	0.97
HDL Cholesterol (mg/dL)	43.91 ± 13.38	47 ± 11.73	43 ± 13.48	42 ± 16.62	0.99	0.61	0.97
GOT (U/l)	20.85 ± 17.56	18.50 ± 18.14	22 ± 18.97	22 ± 7.54	0.99	0.99	0.97
GPT (U/l)	27.50 ± 25.23	23.50 ± 17.50	29 ± 31.91	30 ± 14.16	0.65	0.99	0.97
GGT (U/l)	29 ± 23.04	27.50 ± 18.04	30 ± 25.84	32 ± 23.3	0.65	0.61	0.99
HCT (%)	41 ± 4.03	40 ± 4.09	41.1 ± 3.05	40.5 ± 6.13	0.99	0.99	0.94
Leukocytes (G/L)	7.84 ± 2.63	7.48 ± 2.4	8.1 ± 2.39	7.8 ± 3.7	0.99	0.61	0.97
Neutrophils (G/L)	5 ± 2.44	4.8 ± 2.36	5.15 ± 2.28	5.3 ± 3.2	0.99	0.99	0.99

Statistical significance (ANOVA) is noted with * when *p* < 0.05

### Liver biopsies and liver fibrosis diagnosis

Liver biopsies were performed during laparoscopic surgical bariatric procedures or via ultrasound guided liver biopsy, as previously described [4]. No energy devices were used for collecting the samples since hemostasis was done afterwards when the samples were extracted from the abdomen. Ultrasound (US) guided percutaneous liver biopsy (UPLB) was performed in 10 patients. In all patients, antiplatelet drugs and oral anticoagulation therapy was paused 1 week before UPLB was performed. One experienced physician (> 3000 US-exams and > 100 UPLB) performed the US-examinations with the Philips EPIQ 5® (Philips Corporation, Amsterdam, The Netherlands). UPLB was performed using an 18 G Temno II semi-automatic tru-cut biopsy needle (Cardinal Health, Dublin, Ohio, USA). After UPLB, all patients were monitored for any signs of pain or clinically suspected bleeding by nursing staff over a 6-h period. If no serious complications were evident, all patients would be discharged after the mandatory 6-h observation, a stable blood count and a normal ultrasound examination. All patients were follow-up in 2 weeks to review the results of the histology. All the samples were stored in a sterile container and kept at -80 °C until assayed. Furthermore, NAFLD was confirmed histologically by one independent pathologist.

#### Clinical assessments

Anthropometric measurement of each subject was performed by trained nurses in the morning after fasting for at least 8 h. Body height was recorded to the nearest 0.5 cm and body weight to the nearest 0.1 kg. BMI was defined as body weight (kilograms) divided by the square of body height (meters). Waist circumference was measured in the horizontal plane midway between lowest rib and the iliac crest to the nearest 0.1 cm at the end of a normal expiration repeatedly in men and women by 3 trained nurses on 3 consecutive days. Blood pressure was recorded to the nearest 2 mmHg by a mercury sphygmomanometer with the arm supported at heart level after sitting quietly for 10 min. Fasting plasma glucose was measured after fasting for at least 8 h. A standard oral 75-g glucose tolerance test was performed to measure 2-h postprandial plasma glucose. Hypertension was defined in accordance to the Guidelines of the European Heart Association or if the subject was taking medication for hypertension. Diabetes was diagnosed when fasting plasma glucose was ≥ 126 mg/dL (7 mmol/L), 2-h postprandial plasma glucose ≥ 200 mg/dL (11.1 mmol/L), and HbA_1c_ ≥ 6.5% or if the subject was taking medication for diabetes.

### Biochemical and molecular analyses

#### Plasma parameters

Biochemical analyses including total fasted plasma glucose, cholesterol, high-density lipoprotein (HDL) cholesterol, plasma liver enzymes i.e. aspartate aminotransferase (AST/GOT), alanine aminotransferase (ALT/GPT), gamma-glutamyl transferase (GGT), hematocrit and leukocytes were determined by Cobas 8000, (Roche, Basel, Switzerland) according to the manufacturer´s specification. Elevated liver enzymes were defined as aspartate aminotransferase and alanine aminotransferase. HbA1c was measured by high-performance liquid chromatography (Bio-Rad, Muenchen, Germany) and a Jokoh HS-10 autoanalyzer.

### 16S rRNA gene sequencing and bioinformatic analysis

Genomic DNA was isolated and amplified in a strictly controlled environment at VAIOMER SAS (www.vaiomer.fr; Labège, France) using a stringent contamination-aware approach in the two batches. Total DNA was extracted using a specific protocol designed by VAIOMER SAS to carefully minimize all risks of contaminations between samples, from the experimenters, or environment, as described previously [40–44]. The V3-V4 hypervariable regions of the 16S rRNA gene were amplified by two steps PCR using v1 primers (VAIOMER SAS) and sequenced using MiSeq Reagent Kit v3 (2 × 300 bp Paired-End Reads, Illumina, San Diego, CA, USA), as previously described [41]. The MiSeq sequences, and average of 55,000 raw read pairs per sample and 30,300 read pairs classified in OTUs per sample, were then analyzed using the bioinformatics pipeline established by VAIOMER SAS using FROGS v1.4.0 [44]. Briefly, after demultiplexing the bar-coded Illumina paired reads, single read sequences are cleaned and paired for each sample independently into longer fragments. Operational taxonomic units (OTU) are produced with via single-linkage clustering. The taxonomic assignment is performed to determine community profiles (generated by Blast + v2.2.30 + against the Silva v128 Parc databank restricted to the bacterial kingdom). The clustering algorithm used by FROGS is Swarm. It does not require a fixed clustering threshold for sequence clustering and is set at 97% identity. It is noteworthy that in this study 98.6% of sequences were assigned a taxonomy with either an identity of 100% and > 99% coverage or a coverage of 100% and > 99% identity.

To ensure a low background signal from potential exogenous bacterial contaminations from reagents, experimenters, or consumables, different negative controls were performed. They consist in adding molecular grade water to an empty tube, separately at the DNA extraction and PCR steps. Then, the amplification product is sequenced and performed simultaneously to the analysis of the blood samples. We further run two different batches of sequencing to identify potential experimental contaminants. Both the beta diversity analysis and the qPCR analyses show a clear separation between negative controls and both blood samples and liver samples (Supplementary Fig. 1A-G). The controls performed here confirm that the exogenous bacterial contamination was ten times lower than the tissue signal and could be considered as negligible, having thereby a minimum impact on the taxonomic profiles of the samples, as previously reported in large details [40–44].

### Availability of data and materials

The datasets generated and analyzed during the current study are available in (https://www.biorxiv.org/content/10.1101/2020.12.10.419051v1.full) repository. They were deposited under the primary accession number PRJEB41831 and a secondary number ERP125667 on January 2^nd^ 2023; https://www.ebi.ac.uk/ena/browser/view/PRJEB41831 in the European Nucleotide Archive repository.

### Linear Discriminant Analysis (LDA) Effective Size (LEfSe)

The bacterial profiles were further compared between the three groups using LEfSe pairwise analysis with an alpha cut-off of 0.05 and an effect size cut-off of 2.0. The bacterial diversity analyses (alpha and beta diversity, PCoA and taxonomic composition barplots) were generated using the Phyloseq (v1.14.0), vegan (v2.4.0) and ape (v3.5) packages [45–47] under R environment v3.3.1. LEfSe analysis was performed on the OTU table using the online Galaxy interface to identify bacterial taxa that were differentially abundant in the three liver fibrosis groups [30]. Respective cladograms were generated at the genus taxonomic level. Quantitative plots at the genus taxonomic level were generated in percent of their relative abundance. The graphs show mean data with standard deviation and were generated using GraphPad Prism 6 software [49]. Using the LEfSe algorithm, bacterial taxa that were differentially abundant in analysis of liver fibrosis groups were first identified and tested using the Kruskal Wallis test.

### Beta diversity analysis

The bacterial diversity (alpha and beta diversity) was analyzed and represented using the phyloseq (v1.14.0), vegan (v2.4.0), ape (v3.5), and ggplot2 (3.3.5) packages [45–48] under R environment v3.5.1 with Chao, Inverse Simpson, Simpson and Shannon as indexes. The alpha diversity statistical significance was determined by Wilcoxon rank-test. The beta diversity was calculated for every pair of variables to generate a matrix of distance using Bray–Curtis indexes. From distance matrices, Principal Coordinate Analysis and hierarchical clustering were conducted for graphical representation of the beta diversity. PERMDISP2 procedure was used for the analysis of multivariate homogeneity of group dispersions. The Kruskall-Wallis test was performed to compare abundances across the three groups.

### Multivariate analyses

To visualize the distribution of patients according to their clinical parameters, we performed a Principal Component Analysis (PCA) using FactoMineR (v2.3) and factoextra (v1.0.5) R packages [50, 51]. For the study of 16S rRNA diversity, we first filtered the less abundant OTUs to reduce the noise within the matrix before running the PCA. We eliminated those with abundance < 0.01. We then normalized the OTU table by using the Cumulative Sum Scaling normalization followed by a log transformation, using mixOmics package (v6.6.0) [52]. To explore the metagenomic data and identify the largest sources of variation, another Principal Component Analysis was conducted. Also based on the projection of the dataset into a space of lower dimension and originally designed for regression, we performed a Partial Least Square Discriminant Analysis (PLS-DA) and its sparse version (sPLS-DA) on the normalized OTU table count to predict and select the most discriminative features in the data that help to classify the samples according to the fibrosis variable (package mixOmics).

Since we observed the influence of the metagenomic data on the outcome, we used alternative method of classification such as random forest (package randomForest v4.6–14 [53]). The random forest is built from a multitude of different decision trees and classifiers at training time thereby predicting and storing the predicted target outcome.

### Cluster graphical analyses

The abundance matrix of OTUs can be modeled by a graph using PLNmodels package (v0.9.2 [54]) under R where nodes represent OTUs and edges interactions between each pair of nodes. We developed an analysis in clusters i.e. the L1-spectral clustering, implemented in R, a robust variant of the well-known spectral clustering that aims to detect the natural structures of a graph by taking advantage of its spectral properties. The adjacency matrix modeling the variable associations of the graph is used as an input of the l1-spectralclustering algorithm. Due to the influence of the origin of the cohort on the graphical classification through clusters we applied “fair” technics with k-median clustering objectives (k = 3). We identified k centers and assign each input point to one of the centers so that the average distance of points to their cluster center is minimized. In the fair-variant, the points are colored while the goal is to minimize the same average distance objective ensuring all clusters to have an approximately equal number of points of each color. This technique called “fairtree” and developed in python takes as input the desired number of clusters, the desired cluster balance and the normalized table count [55].

### Functional metagenomic prediction

Shot gun sequencing cannot be performed, in the experimental conditions that we used since. the depth of the sequencing on the host tissue is too small to identify specifically in tissues the metagenome and hence the potential molecular pathways involved. More than 99.9% of the sequences represent the host eukaryotic DNA. Therefore, we intent to infer, from the taxonomic identification i.e. the OTU clustered from the 16S rRNA gene sequence count table data, metagenomic genes and the corresponding biochemical pathways specific for each group using the PICRUSt2 tool [31] version 2.3.0b for each sample. This process included four main steps: 1) The OTU representative sequences were aligned against the PICRUSt2 reference alignment, 2) these metagenomic alignments were imported into the PICRUSt2 reference phylogenetic tree, 3) The biochemical functions were inferred by the hidden state prediction method using this phylogenetic tree. During this inference process, the abundance values of each OTU were normalized to their respective predicted 16S rRNA gene copy numbers and then multiplied by the respective gene counts of the target bacteria, 4) The predicted functions were mapped to the MetaCyc database to determine the minimum set of pathways present in the samples. The resulting core output was a list of enzyme functions (Enzyme Commission numbers) with predicted count data for each sample from step 3 as well as a list of MetaCyc pathways with predicted count data for each sample from step 4.

## Results

### Graphical classification of the clinical variables by principal component analyses

**Fig. 1:**
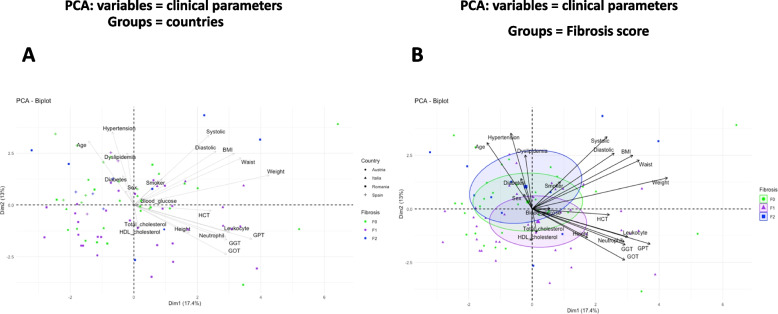
Visualization of clinical variables by principal component analysis according to countries and fibrosis scores. The clinical variables were used as entries for a principal component analysis (PCA). PCA-biplot from package Factoextra and FactomineR of individuals for the first two principal components are shown. They sum up 30.4% of the total variance of the dataset. Patients were grouped by **A**, countries and fibrosis scores (shape and colours) and by **B**, fibrosis scores (green dots = F0, purple triangle = F1, blue square = F2). The vectors corresponding to the clinical variables are shown as arrows

We aggregated together a library of liver biopsies from patients issued from four cohorts of different European countries. We first visualized the distribution of the patients according to the cohorts by performing a Principal Component Analysis using the anthropomorphic and clinical data where the projection of the different clinical variables is represented (Fig. 1A,B). The ellipses calculated for each cohort show some degree of differential distribution suggesting that specific environmental factors have influenced the clinical outcomes. In addition, we could observe some outlier patients from each cohort since they have a highly specific clinical profile.

It is noteworthy that we voluntarily included all anthropomorphic and biochemical data, even if some were redundant and confounding, to remain within the frame of a non-a priory statistical approach. The age, diabetes and hypertension variables were the main drivers of the F2 classification while HDL cholesterol and liver enzymes were drivers for the F1 histological phenotype. These observations are characterized by statistical significance when performing ANOVA tests (Table 1).

### Analyses of the liver bacterial 16S rRNA gene ecology

**Fig. 2:**
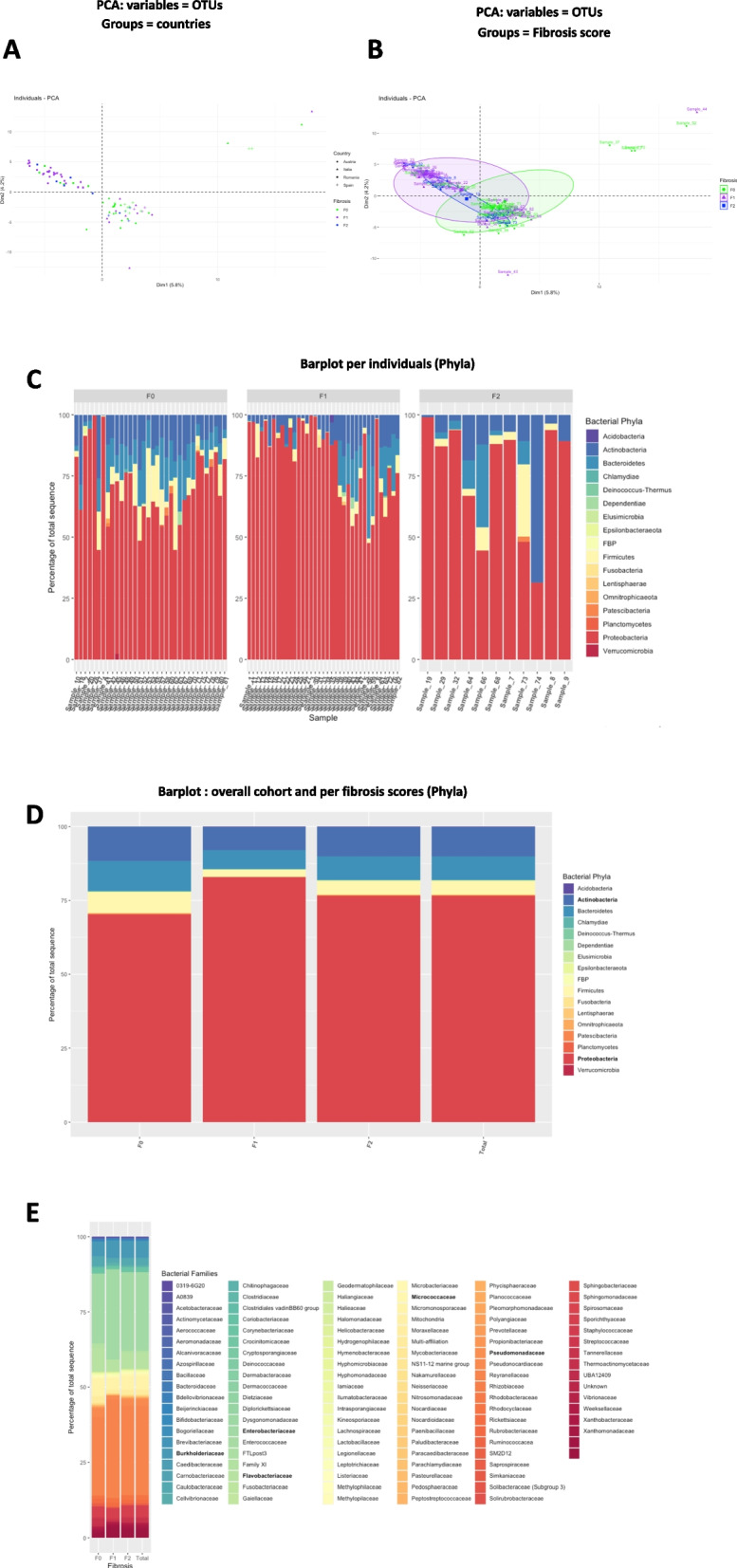
Visualization of liver 16S rRNA gene sequences by principal component analyses according to countries and fibrosis scores. The 16S rRNA gene OTUs sequences were used as entries for a principal component analysis (PCA). PCA-biplot from package Factoextra and FactomineR of individuals for the first two principal components are shown. They sum up 10.0% of the total variance of the dataset. Patients were grouped by **A**, countries and fibrosis scores (shape and colour) and by **B**, fibrosis scores (green dots = F0, purple triangle = F1, blue square = F2). The vectors corresponding to the clinical variables are shown as arrows. **C** Barplot depicting the frequencies of liver microbial composition of each patient at the phylum level depending on their fibrosis stage or **D** as means of the phyla frequencies or **E** the family frequencies for the overall cohort (total) or according to the fibrosis scores (F0, F1, F2)

To identify whether the graphical differences between the three liver fibrosis scores are associated with a discriminant liver bacterial DNA signature, we sequenced the 16SrRNA gene from the liver biopsies. It is noteworthy that only a 16SrRNA targeted metagenomics approach could be performed from tissue biopsies. A shot gun sequencing approach is not doable at a regular depth since almost 99.9% of all sequences are from the host DNA. In addition, we took extreme care in discriminating the potential contaminant bacterial DNA from the environment, including the supplies used, from the tissue specific bacterial DNA sequences. Numerous negative controls were performed (Supplementary Figs. 1 A-D) as well as repeated analyses, as shown (Supplementary Fig. 1 E). The background and individual sequencing data are shown (Supplementary Figs. 1 F,G). We clearly identified that the potential contaminants were 10–100 times lower in amount than the tissue specific bacterial DNA. Therefore, from the tissue specific 16SrRNA sequences we then performed PCA using OTUs as variables in the database. The analysis using countries as groups shows that the Romanian cohort and the Spanish, Austrian, Italian cohorts poorly overlapped suggesting the existence of confounding factors such as the cohort itself (Fig. 2A). Using the liver fibrosis scores as groups and the OTUs as variables we could not clearly graphically discriminate the fibrosis scores (Fig. 2B). The distribution of the patients according to their OTU profiles were too scattered and seemed to be depending upon the largest Romanian cohort. To analyze differently the putative signatures according to the liver fibrosis scores and not the cohort origin, we studied the frequencies of the phylum and family taxonomic levels. The barplot analysis shows first a large degree of heterogeneity between all individuals at the phylum level (Fig. 2C) but still, we identified that the liver microbiota of the overall cohort was composed mostly of Proteobacteria, (> 75%) (Fig. 2D). Group comparisons showed that statistical differences were observed between the F0 and F1 groups for the Proteobacteria, Bacteroidetes phyla (Supplementary Fig. 2 A,B). At the family taxonomic level, the most prominent taxa were the Enterobacteriaceae and the Pseudomonadaceae which accounted for more than 50% of the overall taxa (Fig. 2E). Group comparisons showed that the Flavobacteriaceae and Xanthobacteriaceae families were statistically different when comparing F0 and F1, using a corrected t-test (Supplementary Fig. 2 C,D).

To further identify whether liver fibrosis scores could be characterized by specific signatures we explored indexes of alpha and beta diversity of the 16S rRNA gene sequences in liver tissue. The data show that the differences in abundances at the phylum, and family taxonomic levels observed were also associated with differences of the alpha diversity (Supplementary Fig. 3A,B). Notably, the most standard alpha diversity indexes (Observed, Shannon and Simpson) were significantly different between the F0 and F1 groups at the phylum and family levels, as assessed by the Wilcoxon rank-tests (Table 2). In addition to the alpha diversity, we analyzed the beta diversity and performed a Principal Coordinate Analysis (PCoA) considering distances between variables i.e. sequence similarities (using Bray–curtis distance). The PCoA analyses showed that the F0 group was distant from the two others which suggests a specific 16S rRNA gene signature (Supplementary Fig. 3C,D). It is noteworthy that outlier patients were also detected. Although, when analyzed together the three groups could not be clearly separately classified. We however, ruled out a potential batch effect. To determine if the ellipse centers of the F0 group differs from the ellipse center of the other groups, a Permutational Multivariate Analysis of variance (PERMANOVA) followed by a Kruskall-Wallis test were performed. As the geographical origin of the samples has a prominent influence on the clustering and the microbial profiles, we included this parameter in the PERMANOVA model and found a difference between F0 and F1 groups (*p* < 0.03). Table 1 lists multiple characteristics for the fibrotic patients. We tested their effect on the microbiome using alpha and beta diversity analyses. Along the same line of investigation, we performed different graphical representations such as heatmaps and Venn diagrams.

**Table 2:** means ± SD of different alpha diversity indexes at the family taxonomic level for the three liver fibrosis groups of patients

	**F0**	**F1**	**F2**
**Chao1**	**49.7 ± 16.3**	**48.6 ± 11.5**	**42.49 ± 10.36**
**InvSimpson**	**8.58 ± 3.9**	**7 ± 3.97**	**7.5 ± 4.25**
**Observed**	**40.4 ± 11.31**	**40.89 ± 9.23**	**35.36 ± 6.8**
**Shannon**	**2.46 ± 0.40**	**2.25 ± 0.48**	**2.2 ± 0.61**
**Simpson**	**0.85 ± 0.1**	**0.80 ± 0.12**	**0.82 ± 0.13**

### Identification of specific bacterial signatures

**Fig. 3:**
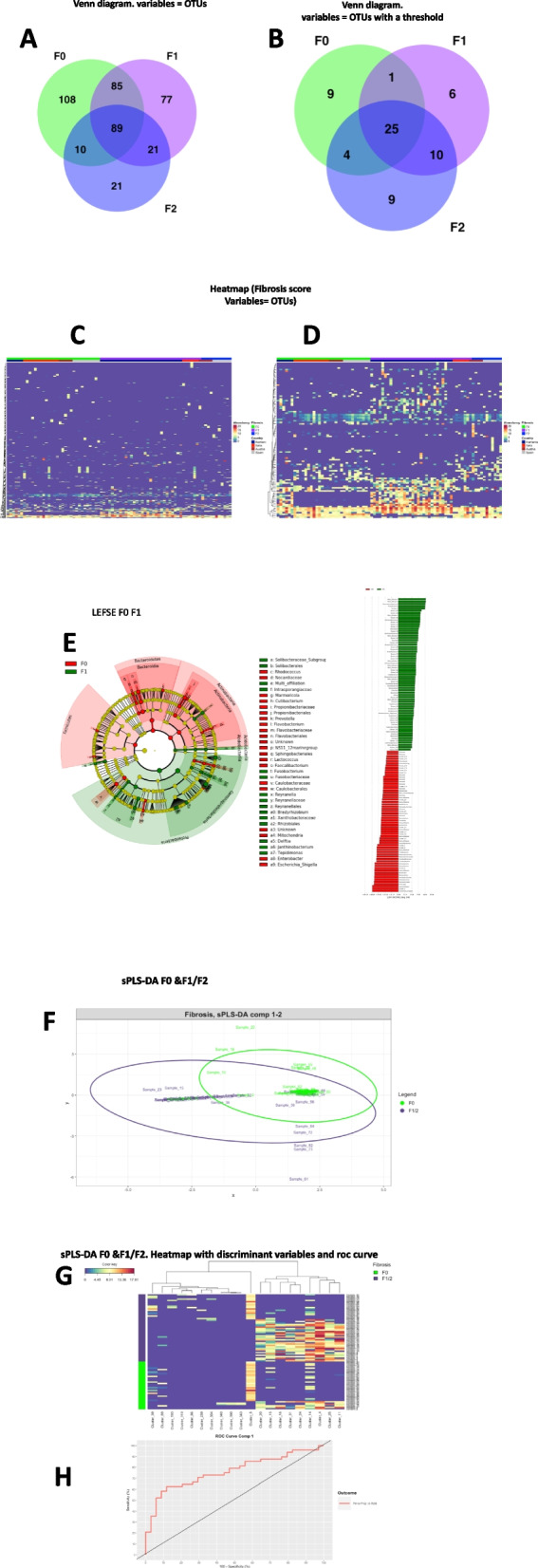
Discriminant analysis strategies of the liver microbiota 16S rRNA gene OTUs according to the fibrosis scores. Venn diagrams where **A** all the 16S rRNA gene taxa or **B** data after removing those extremely rare and with unbalanced distribution within the 3 groups of patients with liver fibrosis, were used as entry variables characterizing the 3 liver fibrosis scores (green = F0, purple = F1, blue = F2). **C** Heatmap of normalized OTU counts according to the 3 groups of patients with liver fibrosis scores and their geographical origin and **D** a corresponding subset of normalized OTU counts with groups of patients fixed. **E** LEfSe cladogram of taxonomic assignments from 16S rRNA gene sequence data of the two liver biopsy fibrosis groups (F0 and F1). The cladogram shows the taxonomic levels represented by rings with phyla at the innermost ring and genera at the outermost ring, and each circle is a member within that level. Taxa at each level are shaded according to the liver fibrosis group in which it is more abundant (*P* < 0.05; LDA score ≥ 2.0). LDA scores are shown on the right panel for each taxon. **F** sPLSDA classification performance on a CSS normalized microbial table count of the F0 versus F1/2 groups of patients. OTUs were labeled as “Cluster_i” with i from 1 to 411 (total number of variables in the normalized abundance matrix). Sample plot, each point corresponds to an individual and is colored according to its fibrosis score (green = F0, purple = F1/2). **G** Clustering Image Map (CIM) of the OTUs selected on each sPLS-DA component with groups of patients fixed. **H** ROC calculated on the predicted scores obtained from the sPLSDA model

To identify the variables that are specific to Fibrosis scores we performed a first Venn diagram on the overall set of variables (Fig. 3A). Eighty-nine variables were common to all groups and considered as the core of the cohort while 21, 77, and 108 OTUs were specific to the F2, F1, F0 groups, respectively. To isolate extremely rare variables and take into account the unbalanced distribution between groups we next considered only OTUs with more than 25% of non-zero counts and an average number of counts per group higher than 150. We then similarly drew a second Venn diagram. We identified 9, 6, and 9 OTUs specific to F2, F1, and F0 scores, respectively (Fig. 3B) and (Table 3). To identify if these specific OTUs could be identified using another approach we generated a heatmap where each OTUs was positioned while the fibrosis scores was fixed (Fig. 3C). We noticed that the frequencies of the majority of OTUs equal 0 or are extremely low (< 0.01%) thereby, most of these variables do not bring information. Similarly, a minority of the variables of high frequencies were common to all liver fibrosis groups and did not provide discriminant information neither. Such OTUs could be considered as the core variable of liver microbiota. Conversely, a subset of OTUs could be considered discriminant since identified from a different heatmap following the removal of the non-informative OTUs (Fig. 3D).

**Table 3:** Identification of specific bacterial signatures (unfair analyses) using corrected t-test

Family	Genus	CSS mean (F0,F1,F2)
**F0**		
Streptococcaceae	Streptococcus	2.25/0.95/1.13
Flavobacteriaceae	Flavobacterium	1.81/1.54/0.03
Moraxellaceae	Acinetobacter	1.14/0.58/0
Ruminuococcaceae	Faecalibacterium	1.50/0.3/0
Microbacteriaceae	Rhodoluna	2.78/1.51/0.51
Sphingomonadaceae	Sphingomonas	0.28/0.6/0.41
Microccoccaeae	Kocuria	2.61/1.32/0.95
Caulobacteriaceae	Caulobacter	2.24/1.34/0.34
Spirosomaceae	Pseudarcicella	1.19/1.24/1.32
**F1**		
Lachnospiraeae	Multi-affiliation	0/0/0.76
Corynebacteriaceae	Corynebacterium	0/0/1.58
Weeksellaceae	Cloacibacterium	1.04/1.27/1.91
Peptostreptococcaceae	Romboutsia	0.57/0.2/0.86
Enterobacteriaceae	Morganella	0.66/1.97/1.68
Burkhoderiaceae	Delftia	0/0.75/3.19
Microbacteriaceae	Clavibacter	0/0/2.07
**F2**		
Pseudomonadaceae	Pseudomonas	0.05/0.33/0
Burkholderiaceae	Janthinobacterium	0.46/1.54/0.88
Intrasporangiaceae	Multi-affiliation	0.26/1.75/0.38
Burkhoderiaceae	Comamonas	0.81/2.71/1.45
Rhodobacteaceae	Paracoccus	1.35/0.87/0
Ferruginibacter	Metagenome	0/0.32/0

Clusters were identified from the overall database prior to applying the fair strategies. The impact of countries is observed as shown in Fig. 2

To refine the identifications of the discriminant bacteria we performed a Linear Discriminant Analysis (LDA) coupled with effect size measurements (Fig. 3E, Supplementary Fig. 4A,B). The data show that most of the discriminant information was extracted when comparing F0 and F1. The Firmicutes, Flavobacteriaceae, Caulobacteraceae and Actinobacteria were specific to the F0 group and the Proteobacteria was specific to the F1 group. On the barplot the taxa enriched in patients with no fibrosis are indicated with a negative score and mild fibrosis enriched taxa are indicated with a positive score. We performed LEFSe between each score and identified much less differences between F1 & F2 suggesting that they could have similar 16SrRNA liver profiles, as suggested in Fig. 2B despite the discriminant clinical variables identified in Fig. 1B.

From these first sets of analyses, the number of patients per liver fibrosis score was too heterogeneous to perform a discriminant analysis (overfitting). As shown on supplementary Fig. 3C there was almost no difference between F1 and F2, therefore, we merged F1 and F2 scores and defined the F1/2 group, increasing hence the number of patients of that group.

To validate the pertinence of such strategy we performed a Partial Least Square Discriminant Analysis i.e. PLS-DA. To select the most discriminant features in the model we used its sparse version sPLS-DA based on a Lasso penalization. The number of variables to be selected per component involved in the visualization is optimized using the leave-one-out cross-validation approach. On the sample plot (Fig. 3F), we observed a slight separation of the two fibrosis scores ellipses compared to the unsupervised PCA. From the most discriminant OTUs selected on each sPLS-DA component, a dissociation between the two groups is visualized using a Clustering Image Map (CIM) technique (Fig. 3 G,H). The graphs show a clear classification of the patients based on the identified discriminant variables. Eventually, we calculated the ROC curve with all discriminant variables. It shows a specificity and sensitivity (0.76) above baseline (0.50, Fig. 3I).

Altogether, some degree of graphical classification of the liver fibrosis score could be observed using the clinical database and the 16SrRNA gene database. However, in both instances the individuals appear to be still distributed across countries. Therefore, to overcome this issue we developed an ad hoc fairness statistical strategy allowing the classification of variables i.e. OTUs independently from the cohort.

### Identification of clusters of cohort-independent 16S rRNA gene associated with different mild scores of fibrosis

**Fig.4:**
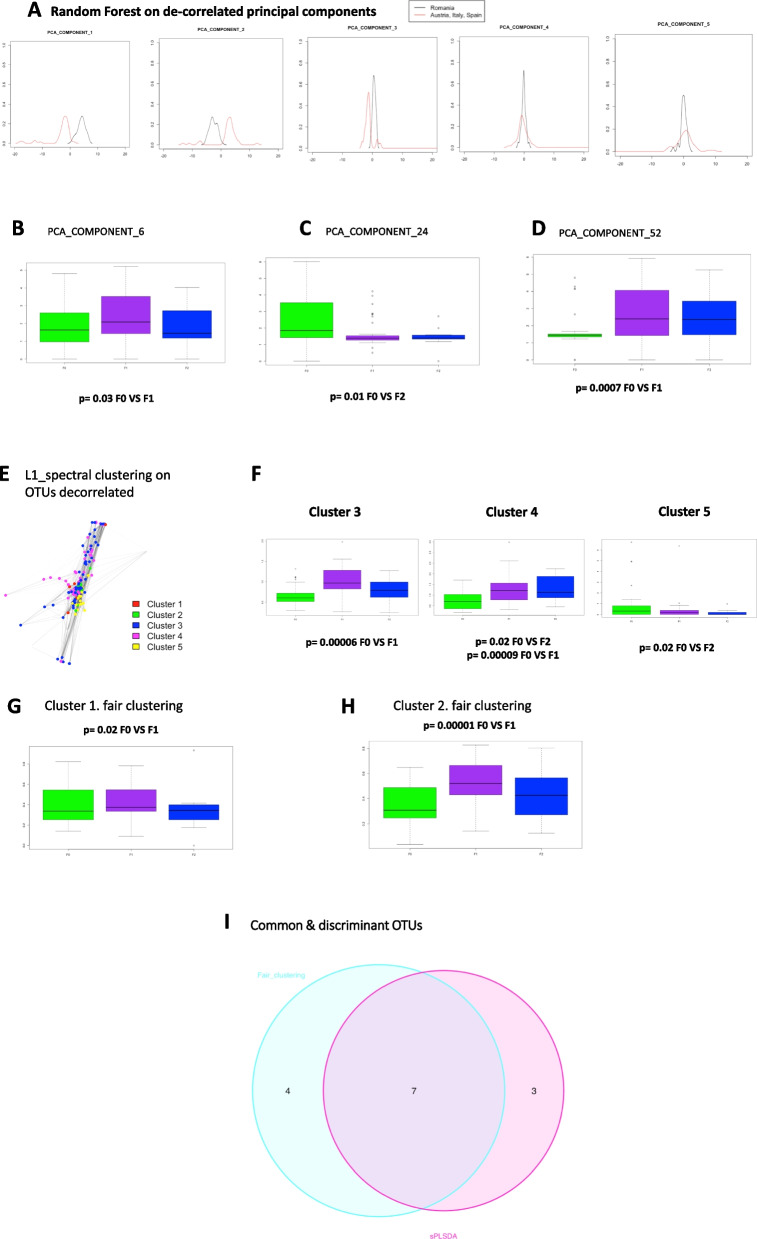
Discriminant analyses of the 16S rRNA gene OTUs variables using fairness strategies. **A** Distribution curves (or densities) of the coordinate of individuals, split into two cohort types (black = Romania, red = the other countries: Italy, Austria, and Spain), when projected on the five first principal components built from the 16S rRNA gene OTUs normalized table count. The non-overlapping plots (for example components 1,2,3) correspond to cohort discriminant components and will be removed from the final analysis to identify the liver fibrosis discriminant variables. Boxplot representing the frequencies of the most significant OTUs contributing to **B** the 6^th^, **C** the 24^th^, **D** the 52.^nd^ principal components for the different groups of liver fibrosis scores (green = F0, purple = F1, blue = F2). t Tests were performed for **B-D**, **F–H**. **E** Graphical representation of the normalized OTU table counts whose nodes are colored according to the 5 clusters identified by the l1-spectral clustering algorithm (red = 1, green = 2, blue, 3, pink = 4 and yellow = 5). **F** Boxplot representing the mean frequencies of the OTUs in cluster 3, 4 and 5, identified by the l1-spectral clustering algorithm, for the different groups of liver fibrosis scores (green = F0, purple = F1, blue = F2). **G**, **H** Boxplot representing the frequencies of OTUs in cluster 1, and 2, identified by fair-tree algorithm, for the different groups of liver fibrosis scores (green = F0, purple = F1, blue = F2). **I** Venn diagram depicting the liver microbial taxonomies of common OTUs identified by standard (sPLS-DA) and fair approaches (fairtree, random forest, l1-spectral clustering) as signatures of low fibrosis scores (blue = sPLSDA, pink = fair algorithms)

In front of these numerous signatures and the influence of confounding factors such as the impact of the cohort itself there is a need to identify clusters of variables specific to each liver fibrosis score but independent from the origin of the cohort. To this aim we considered three different fair approaches on the overall cohorts and then defined clusters of OTU variables independent from the cohort. The first fair approach consists in identifying principal components from the metagenomic dataset as signatures of the cohorts and removing them to generate a new dataset where no components would be cohort sensitive. To this aim we compared the largest cohort i.e. from Romania to the others. As an example, we here represent the five first principal components conditional distributions according to the cohorts (Fig. 4A). The last two are characterized by similar distributions, indicating that some principal components are decorrelated from the geographical origin. Hence, we removed the principal component, which contain less than 20% of the information i.e. the most correlated with the cohorts when the absolute value of Pearson correlation was above the threshold of 0.1. The remaining non-overlapping components are cohort-insensitive and used to identify the variables associated with the specific fibrosis score. Remarkably, more than 78% of the variation from the original data was still included into the selected principal components suggesting that the discriminant information was only marginally affecting our previous results. On this “fair” dataset we applied the standard random forest classification to predict fibrosis scores. From the variable importance plot, indicating the contribution of the variables to classify the data, we selected the 10 most predictive principal components and identified from them 3 significantly associated with the fibrosis scores (Fig. 4B-D).

The second fair clustering approach directly integrates in the model the variables affecting the metagenomic dataset. It selects OTUs which are the most influenced by this variable and removes it from the analysis. The matrix formed by the remaining OTUs is then modeled by a graph and subjected to a spectral clustering algorithm to which we applied an L1 penalty. The nodes represent OTUs and the edges show interactions between each pair of variables (Fig. 4E). Using this novel l1-spectral clustering algorithm we identified 5 clusters of OTUs among which 3 were significantly associated with the liver fibrosis scores (Fig. 4F).

Eventually, we performed the fair clustering approach called “fair-tree” [55]. We used the 16S rRNA gene normalized table count to identify clusters with approximately equal numbers of patients from each cohort. Two of the three clusters found containing respectively 36 and 97 OTUs, were statistically significant when comparing F0 versus F1 scores (Fig. 4G,H).

We summarized all the identified OTUs significantly (t-test) associated with the low scores of fibrosis in (Table 4) and identified their respective taxa. From the fair principal components identified, we considered only the five OTUs contributing the most to each of these components. Then, from the Venn diagram we identified common OTUs signatures of low fibrosis scores from standard (sPLS-DA) and fair approaches (fair-tree, random forest, l1-spectral clustering) (Fig. 4I). Interestingly, from all selected OTUs eight common OTUs were from the same phylum i.e. Proteobacteria (Table 5) suggesting that most of the discriminant information could be due to this phylum. However, there is still most likely some information that this predominant family could be hiding. We therefore set a new mathematical strategy to exemplify the low frequency and meaningful bacteria i.e. the TF-IDF (Term frequency-inverse document frequency) approach.

**Table 4:** Identification of clusters of cohort-independent 16S rRNA gene associated with the different low scores of fibrosis using corrected t-test

Family	Genus	Significance	*P*-value	CSS mean (F0,F1)
**sPLSDA**				
Burkholderiaceae	Ralstonia	F0 VS F1	0.001	1.32/4.19
Xanthobacteraceae	Bradyrhizobium	F0 VS F1	0.001	1.13/5.09
Enterobacteriaceae	Multi-affiliation	F0 VS F1	0.001	1.01/4.66
Pseudomonadaceae	Pseudomonas	F0 VS F1	0.001	0.44/4.2
Enterobacteriaceae	Kluyvera	F0 VS F1	0.005	1.34/4.79
Xanthomonadaceae	Stenotrophomonas	F0 VS F1	0.0005	3.92/7.54
Enterobacteriaceae	Multi-affiliation	F0 VS F1	0.01	1.08/5.54
Pseudomonadaceae	Pseudomonas	F0 VS F1	0.02	1.47/5.53
Corynebacteriaceae	Corynebacterium 1	F0 VS F1	0.05	1.07/0.20
Flavobacteriaceae	Flavobacterium	F0 VS F1	0.001	8.84/3.68
**Fair-tree**				
Pseudomonadaceae	Pseudomonas	F0 VS F1	0.004	1.13/5.09
Enterobacteriaceae	Multi-affiliation	F0 VS F1	0.004	0.44/4.2
Xanthobacteraceae	Bradyrhizobium	F0 VS F1	0.003	3.92/7.54
Xanthomonadaceae	Stenotrophomonas	F0 VS F1	0.05	1.08/5.54
**Fair Random Forest**				
Xanthomonadaceae	Stenotrophomonas	F0 VS F1	0.007	1.01/4.66
Enterobacteriaceae	Multi-affiliation	F0 VS F1	0.004	1.34/4.79
Enterobacteriaceae	Multi-affiliation	F0 VS F1	0.004	1.08/5.54
Enterobacteriaceae	Kosakonia	F0 VS F1	0.01	0.44/2.07
Enterobacteriaceae	Kluyvera	F0 VS F1	0.0007	0.88/2.67
**Fair l1_spectral clustering**				
Pseudomonadaceae	Pseudomonas	F0 VS F1	0.01	0.44/4.2
Xanthobacteraceae	Bradyrhizobium	F0 VS F1	0.005	1.13/5.09
Enterobacteriaceae	Enterobacter	F0 VS F1	0.04	2.59/1.37
Burkholderiaceae	Ralstonia	F0 VS F1	0.03	1.32/4.19
Enterobacteriaceae	Multi-affiliation	F0 VS F1	0.01	1.01/4.66
Enterobacteriaceae	Kluyvera	F0 VS F1	0.04	0.88/2.67
Enterobacteriaceae	Multi-affiliation	F0 VS F1	0.003	1.08/5.54

**Table 5:** Microbial signatures common to all strategies using corrected t-test

Family	Genus	Significance	*p* value	CSS mean (F0,F1)
Pseudomonadaceae	Pseudomonas	F0 VS F1	0.0009	0.44/4.2
Xanthobacteraceae	Bradyrhizobium	F0 VS F1	0.0005	1.13/5.09
Xanthomonadaceae	Stenotrophomonas	F0 VS F1	0.003	1.01/4.66
Enterobacteriaceae	Multi-affiliation	F0 VS F1	0.001	1.01/4.66
Enterobacteriaceae	Kluyvera	F0 VS F1	0.002	0.88/2.67
Burkholderiaceae	Ralstonia	F0 VS F1	0.002	1.32/4.19
Enterobacteriaceae	Multi-affiliation	F0 VS F1	0.0005	1.34/4.79

### Low frequency bacterial 16S rRNA gene contains classifying information

**Fig. 5:**
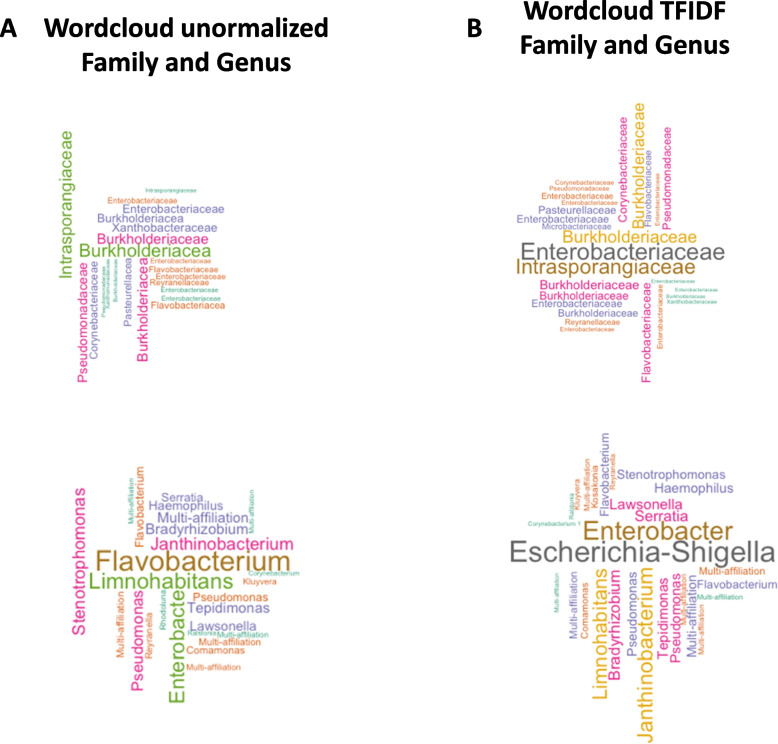
Identification of clusters by wordclouds representation with or without TFIDF normalization. Wordclouds representing taxa of all significant bacteria according to **A,** their frequencies at Family and Genus level or **B**, after TFIDF normalization at Family and Genus level. The size of the name of bacteria is proportional to the frequency of the cluster in the cohorts

From the table counts of all significant OTUs detected we generated a “word-cloud” (Fig. 5A, B) to visualize the most abundant TF-IDF transformed OTU counts, regardless of the fibrosis scores when compared to the most abundant CSS transformed OTU counts. Cluster 2 emerged as the most important discriminant OTU (taxonomic identifaction = Bacteria|Proteobacteria|Gammaproteobacteria|Enterobacteriales|Enterobacteriaceae|Escherichia-Shigella) further confirming the important amount of information contained in the Proteobacteria phylum (Table 5).

Based on the identified specific signatures the next step was to generate hypotheses regarding their potential mode of action to the induction at early only of liver fibrosis. We therefore performed predicted functional metagenomics analyses using PICRUST2 software.

### Predicted functional metagenomics pathways

**Fig. 6:**
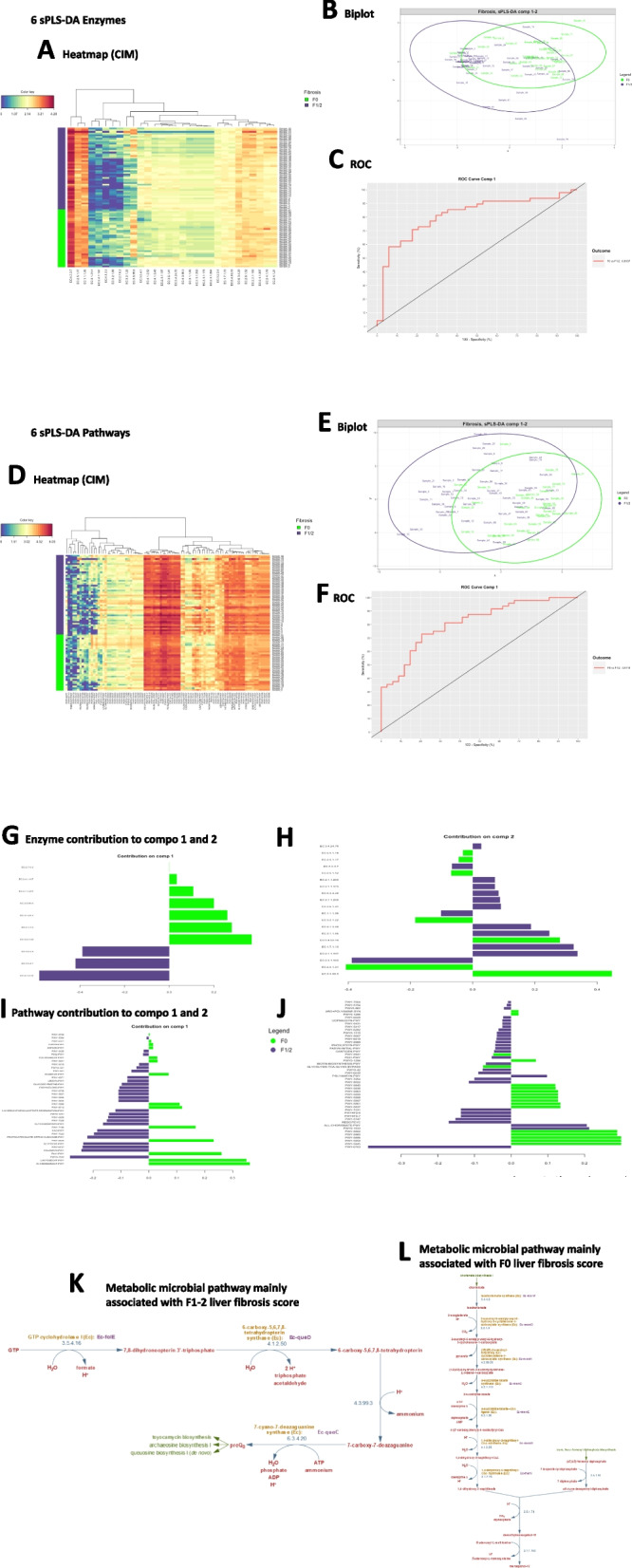
Predicted functional metagenomics analyses of discriminant enzymes and according to the fibrosis score. **A,D** Heatmap (Clustering Image Map (CIM)), **B,E** Sample plot, each point corresponds to an individual and is colored according to its liver fibrosis score (green = F0, purple = F1/2), **C,F** ROC classification performances of **A-C** enzymes, and **D-F** pathways, on a CSS normalized enzyme table count of the F0 versus F1/2 groups of patients. **G-I** Loading plot representing the contribution of each enzyme (**G,H**), and pathways (**I,J**) selected to build the first and second components (green = F0, purple = F1/2). **K,L** main metabolic pathways from the MetaCyc database identified from the Loading plots for the **K** F1-2 and **K** F0 liver fibrosis scores

To identify the pathways and enzymes involved at the onset of liver fibrosis, we run predicted functional metagenomics algorithms based on the fairness-selected bacterial taxa. The heatmap shows clusters of enzymes that are associated with the F0 vs F1-2 fibrosis scores (Fig. 6A). Eventually, sPLS-DA showed also a clear discrimination between the F0 vs F1-2 fibrosis scores. To evaluate the accuracy and sensitivity of our analyses as potential diagnostic tool, we drew a ROC and quantified the urea under curve with a score of 81.4% of accuracy (Fig. 6B,C). We performed a similar analysis on pathways and showed that specific clusters were also discriminately associated with the fibrosis scores. The score of accuracy was of 81.2% (ROC curve) (Fig. 6D-F). We then represented and listed all selected enzymes and pathways highly expressed in the two major discriminant components (Fig. 6G-J) and (Table 6). Three pathways were highly and negatively associated with the F1-2 liver fibrosis score when compared to the F0. We identified from the MetaCyc database [55] that the preQ_0_ biosynthesis (PWY-6703), specific to Enterobacteriaceae such as E. coli, is involved notably in the synthesis of tetrahydrofolate and a class of nucleoside analogues that often possesses antibiotic, antineoplastic, or antiviral activities [32, 33] (Fig. 6K). In addition, two other pathways related to glucoryranose (PWY 6737) and glycogen (GLYCOCAT-PWY) degradation were identified probably providing energy to the main preQ_0_ biosynthesis pathway. On the other hand, six major metabolic pathways were positively associated with the F0 score from both components. One involves the glycolysis and pentose phosphate pathway (PWY-6629), while the 5 others are all involved in the menaquinones and demethylmenaquinones pathways (Fig. 6L). The low-molecular weight lipophilic components of the cytoplasmic membrane are considered vitamin K_2_ components that are found in most aerobic Gram-positive bacteria. They are the main quinones which behave as a reversible redox component of the electron transfer chain, mediating electron transfer between hydrogenases and cytochromes. Altogether the functional metagenomics prediction suggests that gram negative bacteria from the Proteobacteria family composed of preQ_0_ biosynthesis and glycolytic pathways are signature of F1-2 fibrosis scores while the vitamin K_2_ biosynthesis pathway from gram negative bacteria, such as Actinobateriaceae [34, 35], would be a specific signature of the F0 liver fibrosis score.

**Table 6:** Identification of principal enzymes and pathways contributing to the first sPLSDA’s first component and signatures of low score of fibrosis

	**Name**	**Function**
**Enzymes**	EC:4.1.2.52	4-hydroxy-2-oxoheptanedioate aldolase
	EC:3.5.4.1	Cytosine deaminase
	EC:3.2.2.4	AMP nucleosidase
	EC:4.1.3.3	N-acetylneuraminate lyase
	EC:3.1.21.4	Type II site-specific deoxyribonuclease
	EC:3.2.1.89	Arabinogalactan endo-beta-1,4-galactanase
	EC:3.5.99.6	Glucosamine-6-phosphate deaminase
	FAO-PWY	fatty acid &beta,-oxidation I
	PROTOCATECHUATE-ORTHO-CLEAVAGE-PWY	protocatechuate degradation II (ortho-cleavage pathway)
**Pathways**	GLYCOCAT-PWY	glycogen degradation I (bacterial)
	PWY-6737	starch degradation V
	PWY-7323	superpathway of GDP-mannose-derived O-antigen building blocks biosynthesis
	COLANSYN-PWY	colanic acid building blocks biosynthesis
	LACTOSECAT-PWY	lactose and galactose degradation I
	PWY-6629	superpathway of L-tryptophan biosynthesis
	GLCMANNANAUT-PWY	superpathway of N-acetylglucosamine, N-acetylmannosamine and N-acetylneuraminate degradation
	P441-PWY	superpathway of N-acetylneuraminate degradation
	PWY0-1533	methylphosphonate degradation I

## Discussion

We here report a mathematical approach to identify a bacterial 16S rRNA gene signature in liver tissue and corresponding putative biochemical pathways in patients with low scores of fibrosis therefore, at the onset of the disease. Our main finding is that even low scores of fibrosis (F0 vs F1-2) can be classified by biomarkers from the Proteobacteriaceae family within the liver. The second observation is related to the importance of cohort heterogeneity in term of size and data variability which could be major confounding factors that must be taken into account in multi-centric clinical trials or database. We here present a mathematic approach that could help solving this major and common issues.

A gut metagenomics signature of liver fibrosis in humans has been recently described, suggestive of its causal role in the disease [21]. However, such patients where mostly characterized by a high score of liver fibrosis questioning the putative causal role of the liver microbiota at the onset of the disease. We here focused our attention on low scores of liver fibrosis to putatively identify causal factors. We identified mostly sequences from the 16SrRNA gene from gram negative bacteria and notably from the Proteobacteria phylum as signatures of the F1-2 liver fibrosis scores. Among the families, the Proteobacteriaceae, Flavobacteriaceae, and Propionibacteriaceae were discriminating the low fibrosis scores from each other’s. It is noticeable that they all synthesize LPS, a dramatically inflammatory molecule suggesting a pathophysiological role in development of liver fibrosis, probably via the maintenance of a certain degree of immune vigilance. We further refined our analyses and mostly selected the Enterobacteriaceae family from the Proteobacteria phylum suggesting that the liver proinflammation observed during fibrosis would be due or associated with genera from the Enterobacteriaceae family [10]. Moreover, the Micrococcaceae and the Moraxellaceae encompass numerous genera, notably the Arthrobacter and Acinetobacter, that could be as well involved. It is noticeable that we also identified bacterial DNA that could be considered as contaminants since usually from the environment. However, we are living and in closed contact with a complex environment to which we are continuously exposed i.e., the exposome. It is hence physiologically and ecologically understandable that bacteria from the environment could be identified in the host as commensals or saprophytes. Therefore, to ensure that the identified bacterial DNA is not from potential contaminants, as currently found in reagents and materials, we ran numerous technical control samples, as outlined in the method section. We selected signals largely above the background to rule out potential environmental contaminants. From the selected sequences some still remain unusual and would require more investigations to understand their meaningfulness.

The mechanisms through which the gram negative bacteria identified could induce inflammation might be linked to the unique structures of their LPS or peptidoglycans [36]. Furthermore, since such bacteria are motile with flagella, one could also contemplate that the flagella proteins are involved in the liver fibrosis process. However, data report that the TLR5 receptor of flagellin is rather associated with protection against metabolic syndrome, putatively ruling out this hypothesis [37]. To raise potential working mode of action hypotheses we ran a predicted functional metagenomics algorithm (PICRUSt2). It is important to draw the attention of the reader that the following discussion is purely hypothetical, based on genomic assignments which do not correspond to molecular identifications from biochemical quantifications. By inferring from the bacterial genome potential biochemical pathways we identified the preQ_0_ biosynthesis pathway as a signature of F1-2 fibrosis scores. Such pathway is notably identified from gram negative bacteria families such as Proteobacteriaceae [32, 33]. Conversely, the menaquinones and demethylmenaquinones pathways involved in K12 vitamin synthesis were the signature of the F0 score. They are notably produced by the gram positive Actinobacteriaceae such as Bacillus subtilis [35], therefore coherence with our metagenomics findings.

A major hurdle that one can come across when aggregating different cohorts altogether is related to the heterogeneity of the size of the groups and of the diversity of the variables considered. Regarding invasive analyses such as liver biopsies the group size at completion of the inclusions could be different from what predicted during the calculation of power of the trial. Eventually, the distribution of the variables to be studied could be highly heterogeneous for a given disease. Altogether, we here faced several statistical challenges which are linked to liver fibrosis as a disease linked to microbiota. The first major step preceding the microbial analysis is a prefiltering step. We removed OTUs with counts frequencies across all samples below 0.01%, as recommended in [57]. We then used an adapted script to normalize the data to deal with their sparse nature. The package Mixomics [38] used for this study recommends CSS normalization on sparse OTU table counts that could prevent the bias included in the TSS normalization. In addition, it includes multivariate methods for microbiome studies and addresses its limits. In addition, we observed a strong impact of the cohort of origin since the largest cohort from Romania could discriminate the patients from the others based on the 16S rRNA gene OTU variables. The patients could even be classified by cohort when we used the clinical data as entries showing that this issue also has to be taken into account when analyzing the data. Mathematical approaches to overcome this issue are currently being developed however, little has been done regarding the handling of the 16S rRNA gene data now widely used by the scientific community that addresses the role of microbiota on diseases and notably liver diseases. Therefore, we here developed several approaches of fairness to overcome the classical impact of the origin of the cohort.

Off notes, we noticed that two patients from the F1 groups were distributed within the F2 group. This ectopic distribution could be due to the extreme BMI (> 55) featuring a specific clinical phenotype. Conversely, a patient from the F2 group was associated with the F0/F1 distribution. This patient was characterized by his young age (< 40 years old) while the mean age of the F2 group was of 54 years old.

To precisely identify biomarkers of liver fibrosis we performed sets of discriminant analyses. As a preliminary analysis we performed PCoA since better adapted than PCA to dissimilar and sparse data. We then followed our approach by performing a sPLS-DA to identify subsets of 16S rRNA gene that are discriminatory for the liver fibrosis scores. PLS-DA aims to classify a data set according to the values of a qualitative variable by maximizing the covariance between linear combinations of the observed variables and the qualitative outcome. The sparse version, on the other hand, delivers variables per each component, only selected in the OTU dataset, that are the most discriminatory for the liver fibrosis scores. We focused our attention on the identification of the OTU frequencies within and across each group of patients and on the understanding of the importance that OTUs carry within and across the cohorts. We found that the data set is mostly populated by a few high frequency OTUs. However, beside the level of information gained form this approach with overrepresented OTUs we cannot rule out that some more information could be obtained from OTUs rarely represented. Therefore, some information could be hidden in the low frequency OTUs. To test this hypothesis, we introduced a new normalization approach called TF-IDF [39] originally developed for text mining, to attenuate the effects of the high frequency OTUs in the data set. Consequently, we identified a few more OTUs.

## Conclusion

The first evidence of the existence of a liver microbiota opens alternate routes for novel therapeutic strategies since specific bacteria could be involved in the process of liver fibrosis. However, to generate information which could serve as a substratum to reach this aim, we here adapted predicted metagenomics and mathematical approaches to the original and novel nature of the tissue metagenomics data set. We here found that these data are constituted of high heterogeneity variables which are dominated by a few high frequency taxa such as Proteobacteria, signature of F1-2 liver fibrosis scores, and Actinobacteria/Firmucutes, signature of F0 liver fibrosis scores. These major taxa are masking information residing in the lower frequency taxa. Predicting metabolic pathways from selected 16S rRNA gene-based taxa revealed a potential role of folate metabolism in F1-2 liver fibrosis scores while a role of vitamin k12 biosynthesis was characterizing F0 liver fibrosis score. Altogether, the combined use of metagenomics, sPLS-DA, TF-IDF and fairness strategies appeared useful since we identified signatures specific to the lower scores of liver fibrosis i.e. at the onset of the disease.

## Supplementary Information

**Additional file 1.**

## Data Availability

The datasets generated and analyzed during the current study are available in (https://www.biorxiv.org/content/10.1101/2020.12.10.419051v1.full) repository. They were deposited under the primary accession number PRJEB41831 and a secondary number ERP125667 on January 2^nd^ 2023; https://www.ebi.ac.uk/ena/browser/view/PRJEB41831 in the European Nucleotide Archive repository. MiSeq 16S rRNA gene sequences were deposited under the primary accession number PRJEB41831 and a secondary number ERP125667 on December 9^th^ 2020 with a release date on the 31^st^ of December 2021.

## Abbreviations

NAFLD: Non-alcoholic fatty liver disease
PCA: Principal Component Analysis
sPLS-DA: Sparse partial least square – discriminant analysis
TF-IDF: Term frequency-inverse document frequency
HDL: High-density lipoprotein
AST: Aspartate aminotransferase
ALT: Alanine aminotransferase
GGT: Gamma-glutamyl transferase
LDA: Linear Discriminant Analysis
LEfSe: Effective Size
OUT: Operational taxonomic units
MDS: Multiple Dimension Scale
PERMANOVA: Permutational Multivariate Analysis of variance
ANOVA: Analysis of variance
CIM: Clustering Image Map
