# Supplementary material for: Human liver microbiota modeling strategy at the early onset of fibrosis
Source: BMC Microbiol. 2023 Jan 30;23:34. doi: 10.1186/s12866-023-02774-4 (PMC9885577; doi:10.1186/s12866-023-02774-4)
Supplement: Supplementary file 1 — Additional file 1. [file 12866_2023_2774_MOESM1_ESM.pptx]

## Slide 1
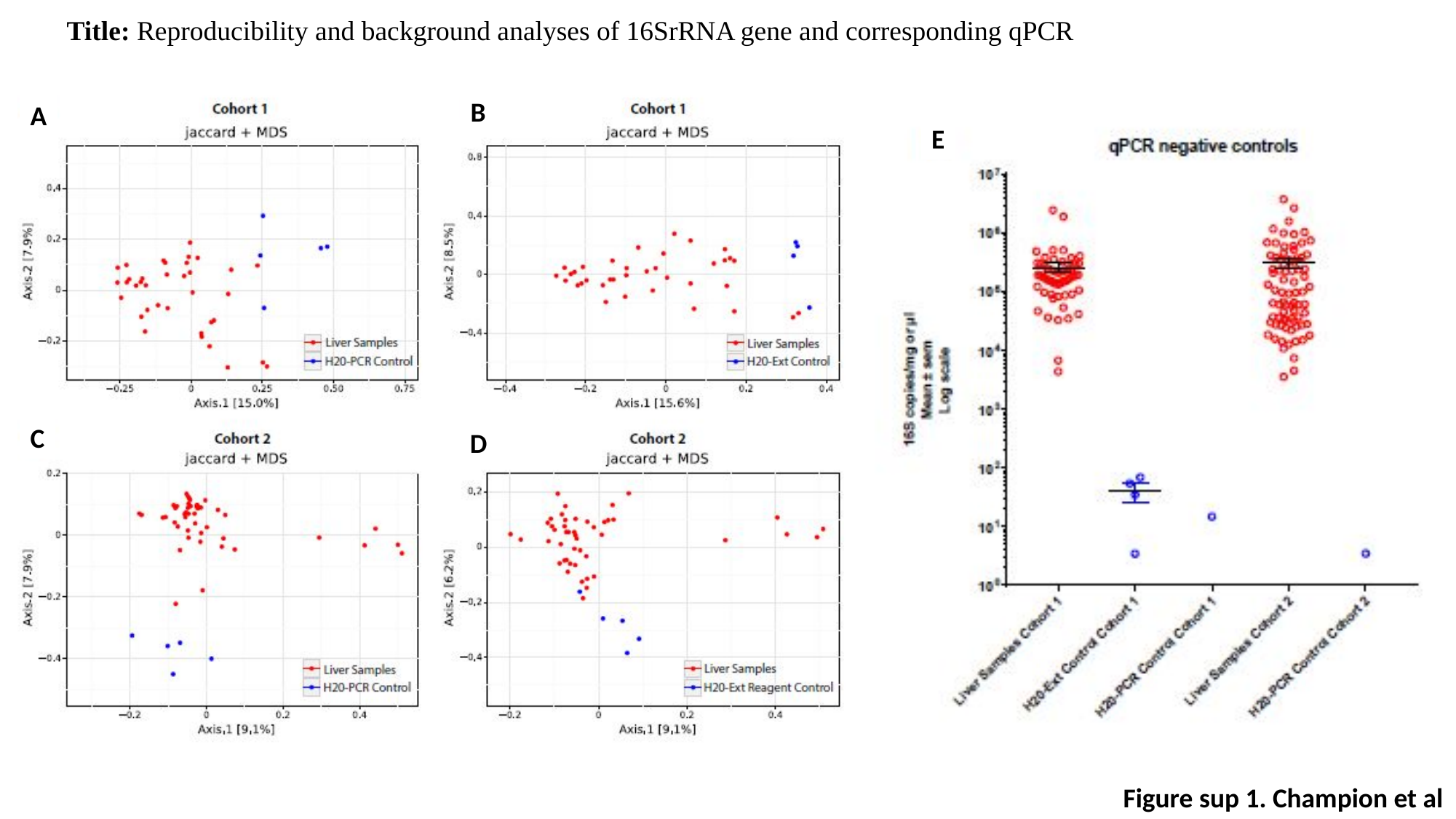

Title: Reproducibility and background analyses of 16SrRNA gene and corresponding qPCR
B
A
E
C
D
Figure sup 1. Champion et al

## Slide 2
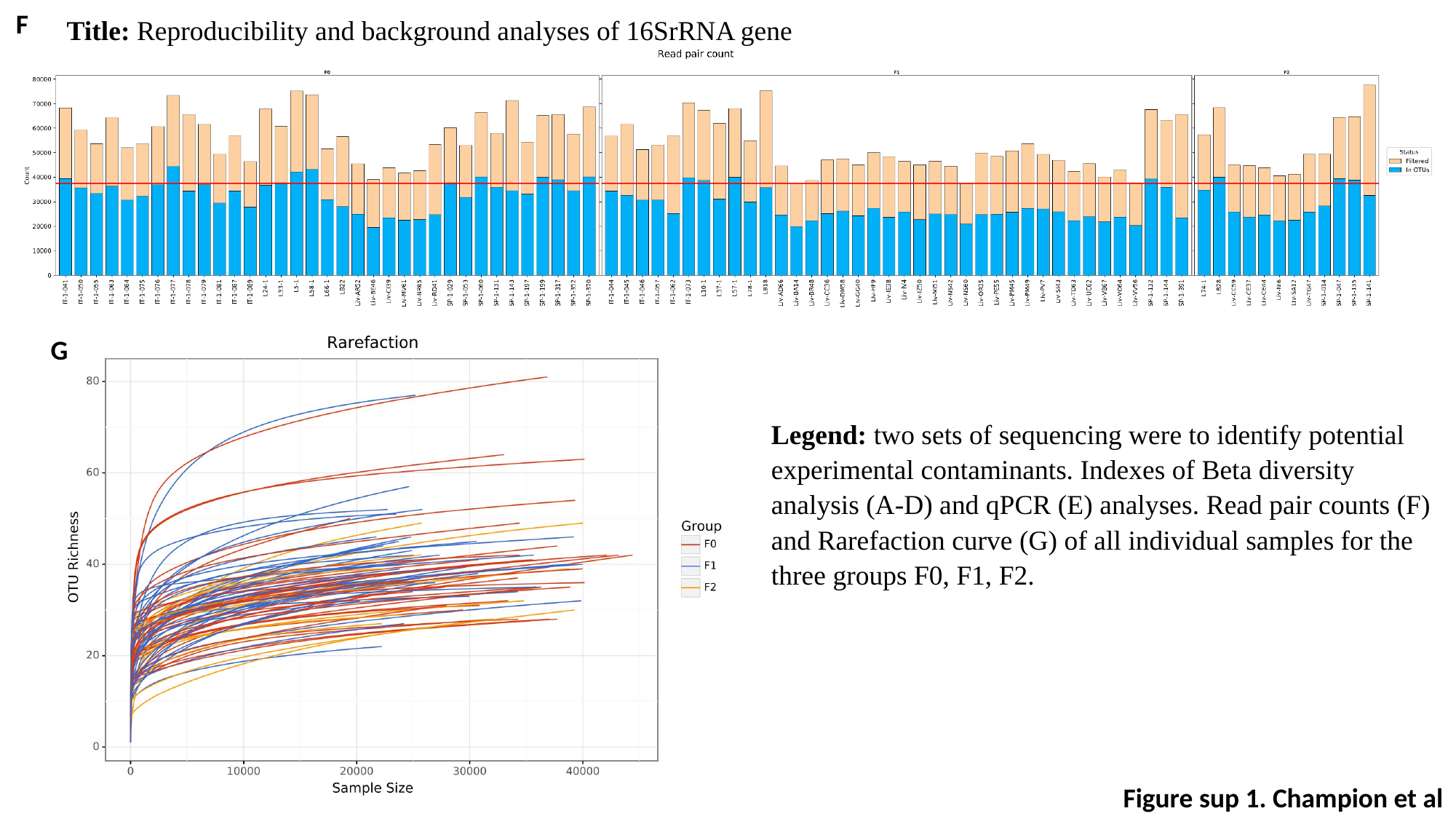

F
Title: Reproducibility and background analyses of 16SrRNA gene
G
Legend: two sets of sequencing were to identify potential experimental contaminants. Indexes of Beta diversity analysis (A-D) and qPCR (E) analyses. Read pair counts (F) and Rarefaction curve (G) of all individual samples for the three groups F0, F1, F2.
Figure sup 1. Champion et al

## Slide 3
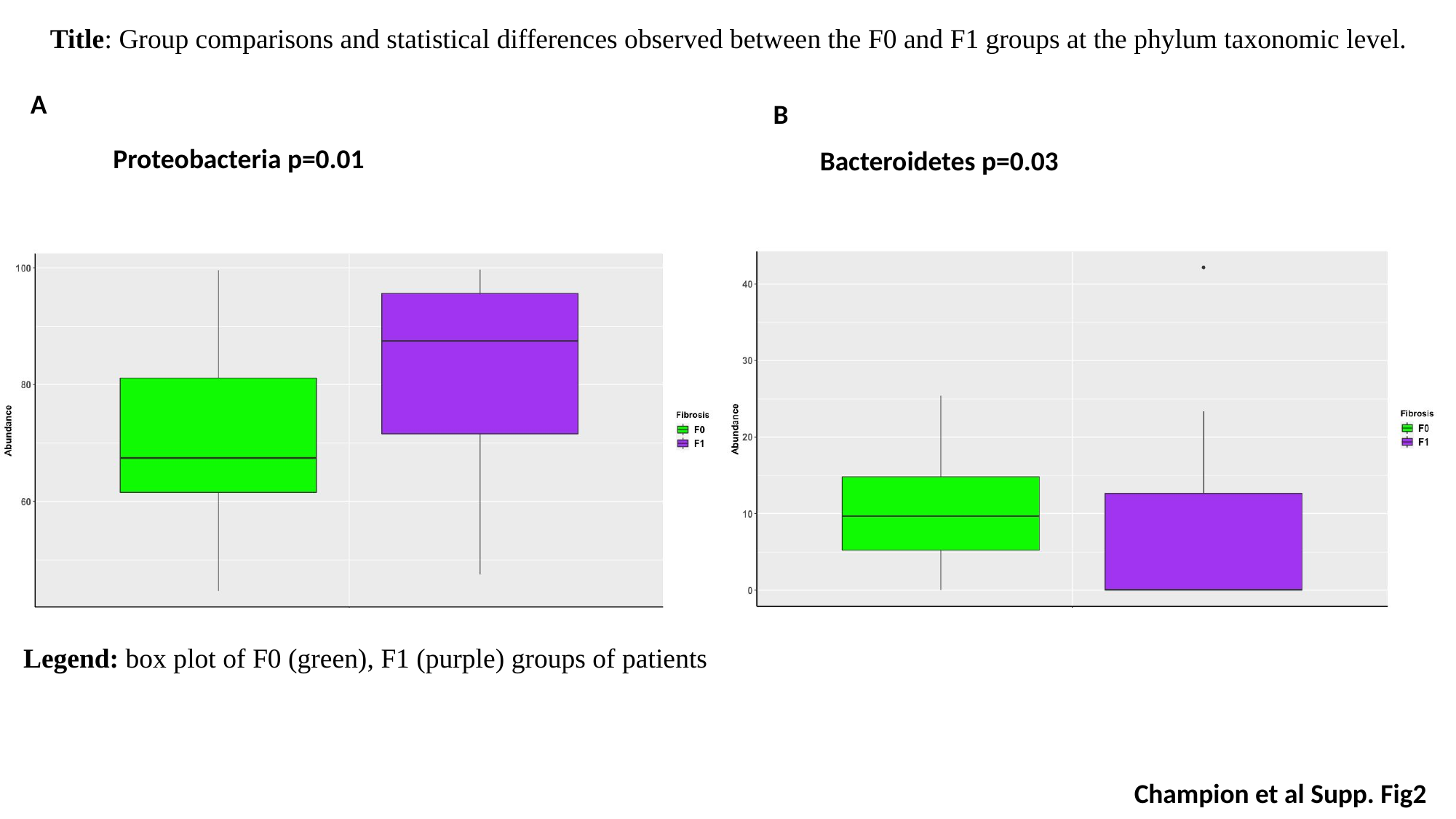

Title: Group comparisons and statistical differences observed between the F0 and F1 groups at the phylum taxonomic level.
A
B
Proteobacteria p=0.01
Bacteroidetes p=0.03
Legend: box plot of F0 (green), F1 (purple) groups of patients
Champion et al Supp. Fig2

## Slide 4
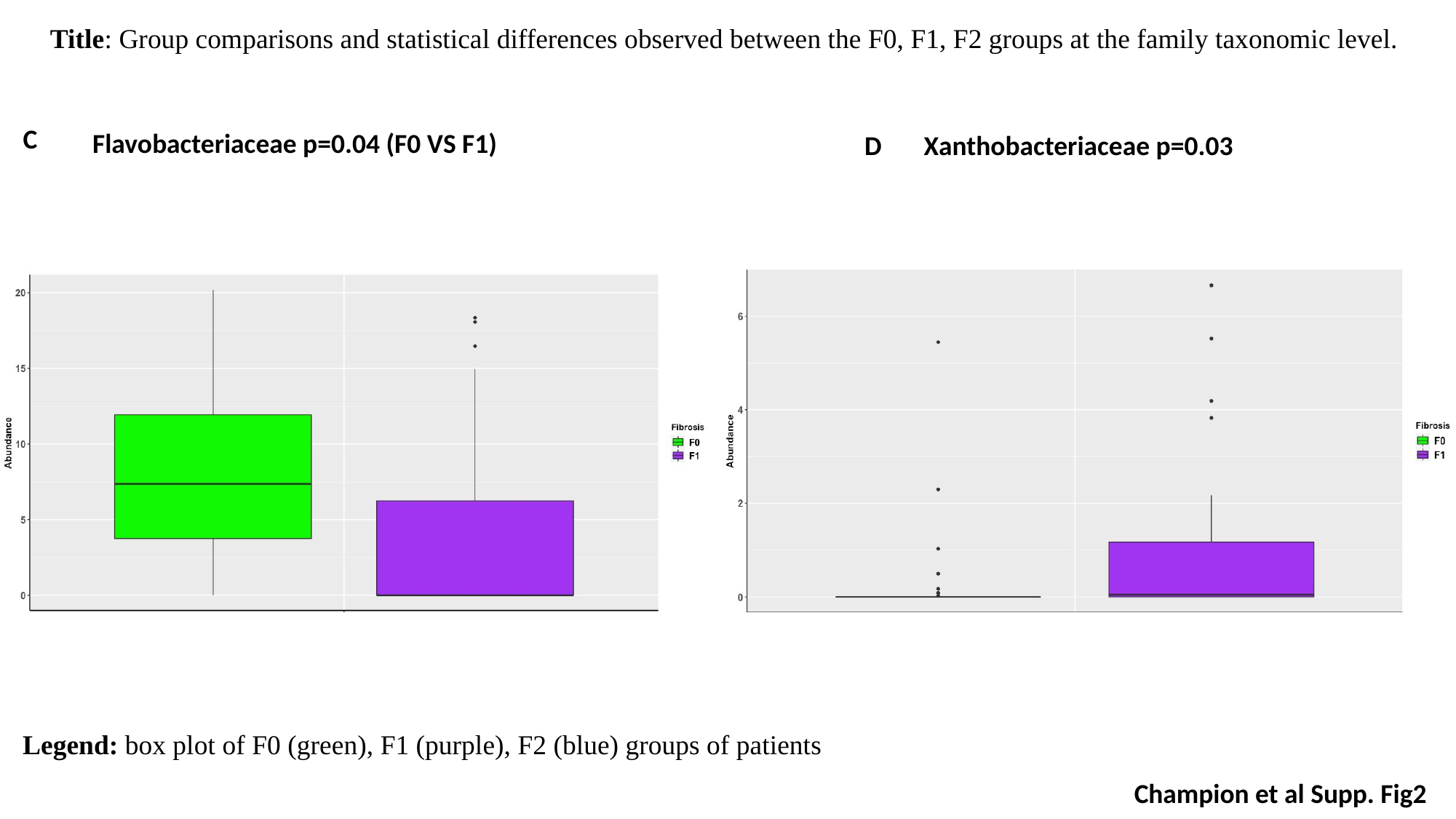

Title: Group comparisons and statistical differences observed between the F0, F1, F2 groups at the family taxonomic level.
C
Flavobacteriaceae p=0.04 (F0 VS F1)
D
Xanthobacteriaceae p=0.03
Legend: box plot of F0 (green), F1 (purple), F2 (blue) groups of patients
Champion et al Supp. Fig2

## Slide 5
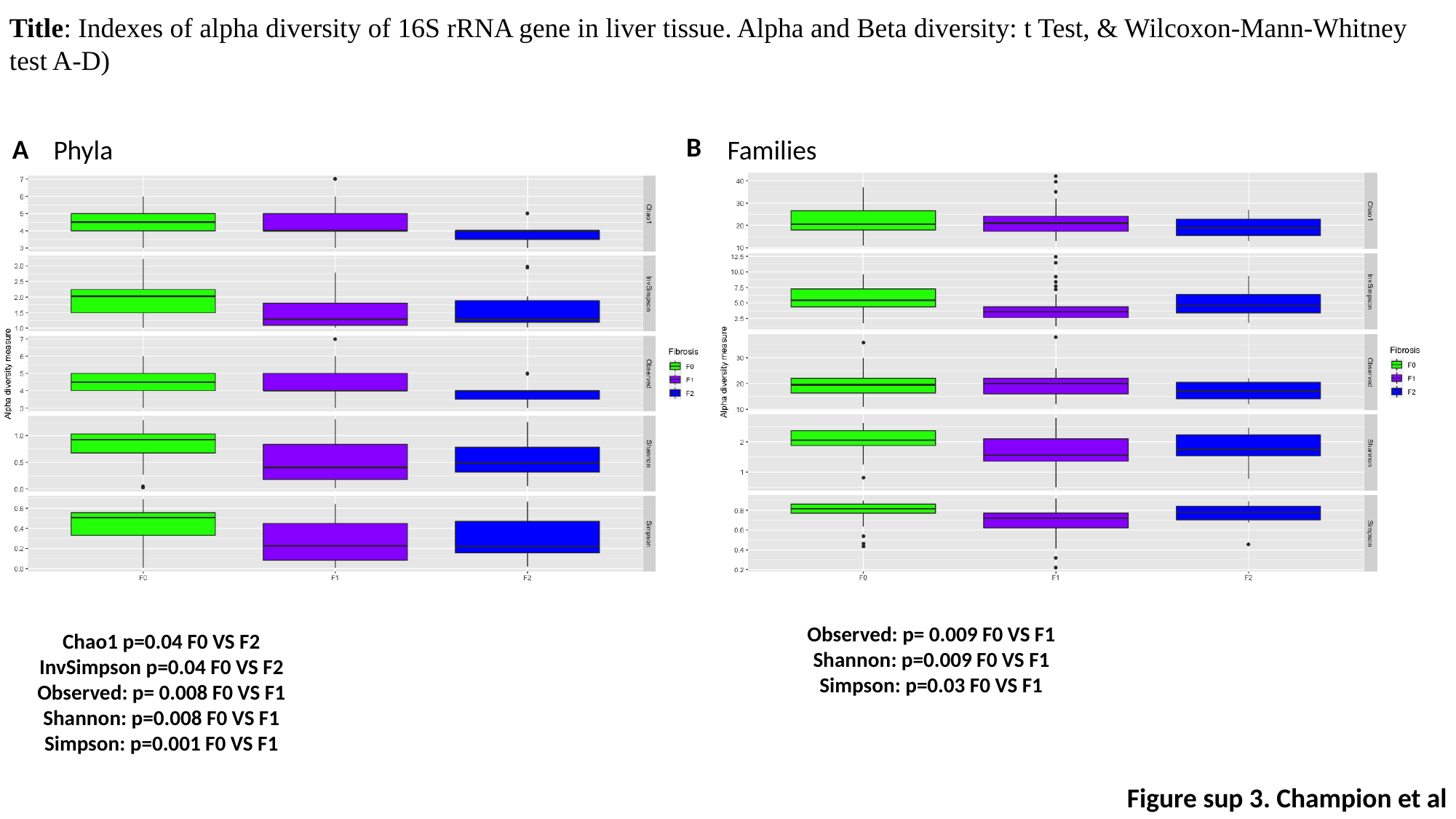

Title: Indexes of alpha diversity of 16S rRNA gene in liver tissue. Alpha and Beta diversity: t Test, & Wilcoxon-Mann-Whitney test A-D)
B
A
Phyla
Families
Observed: p= 0.009 F0 VS F1
Shannon: p=0.009 F0 VS F1
Simpson: p=0.03 F0 VS F1
Chao1 p=0.04 F0 VS F2
InvSimpson p=0.04 F0 VS F2
Observed: p= 0.008 F0 VS F1
Shannon: p=0.008 F0 VS F1
Simpson: p=0.001 F0 VS F1
Figure sup 3. Champion et al

## Slide 6
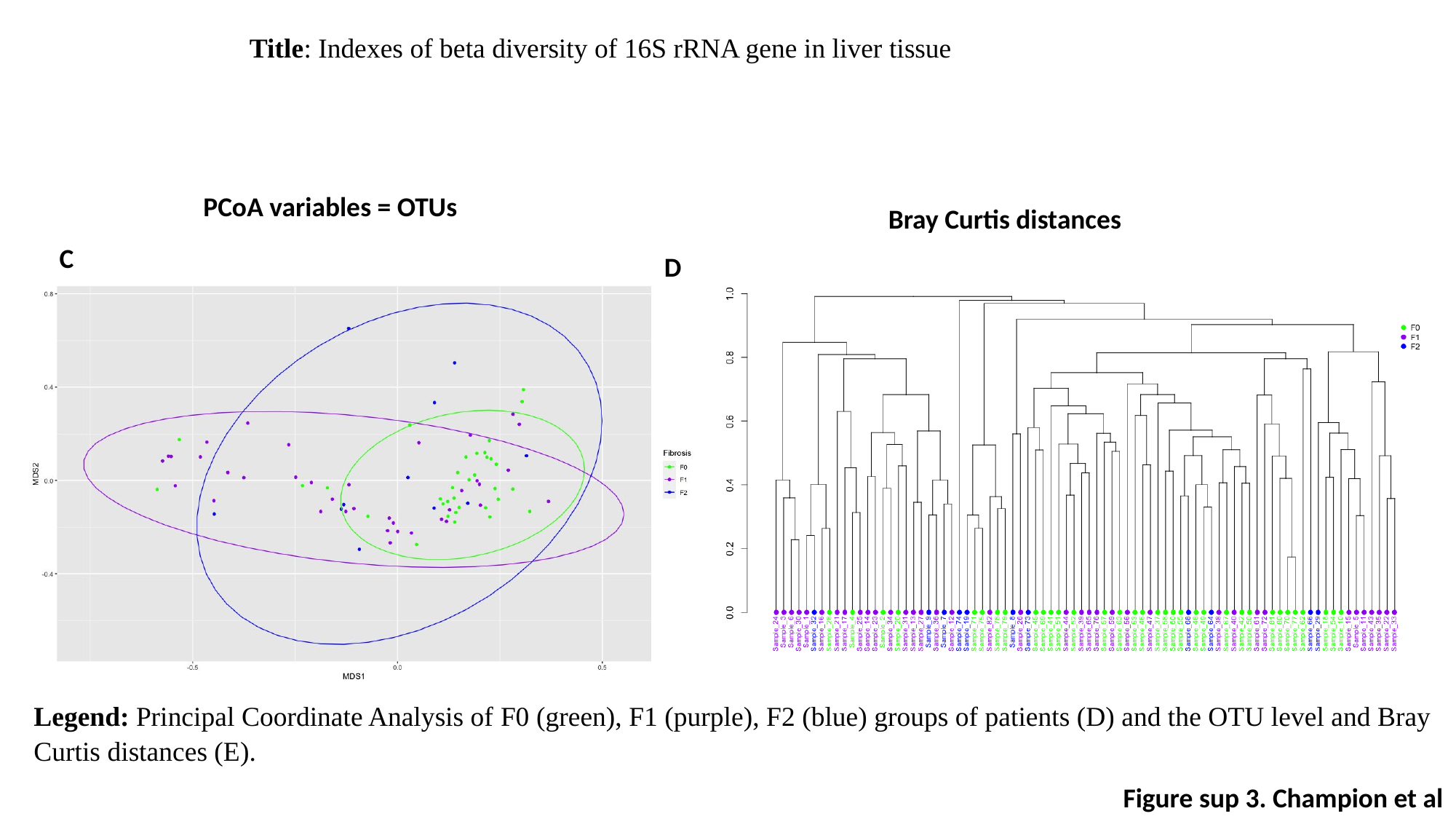

Title: Indexes of beta diversity of 16S rRNA gene in liver tissue
PCoA variables = OTUs
Bray Curtis distances
C
D
Legend: Principal Coordinate Analysis of F0 (green), F1 (purple), F2 (blue) groups of patients (D) and the OTU level and Bray Curtis distances (E).
Figure sup 3. Champion et al

## Slide 7
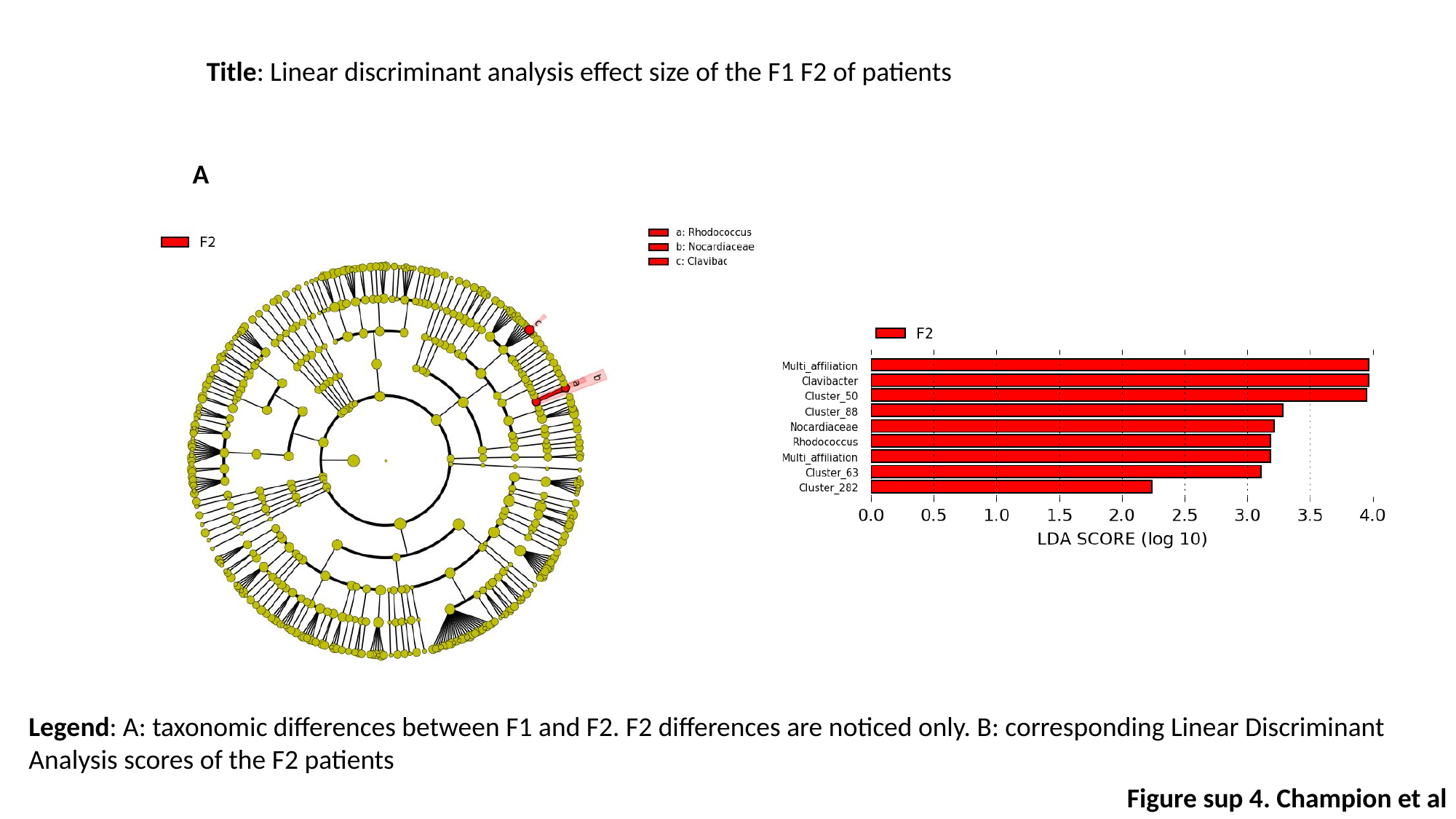

Title: Linear discriminant analysis effect size of the F1 F2 of patients
A
Legend: A: taxonomic differences between F1 and F2. F2 differences are noticed only. B: corresponding Linear Discriminant Analysis scores of the F2 patients
Figure sup 4. Champion et al

## Slide 8
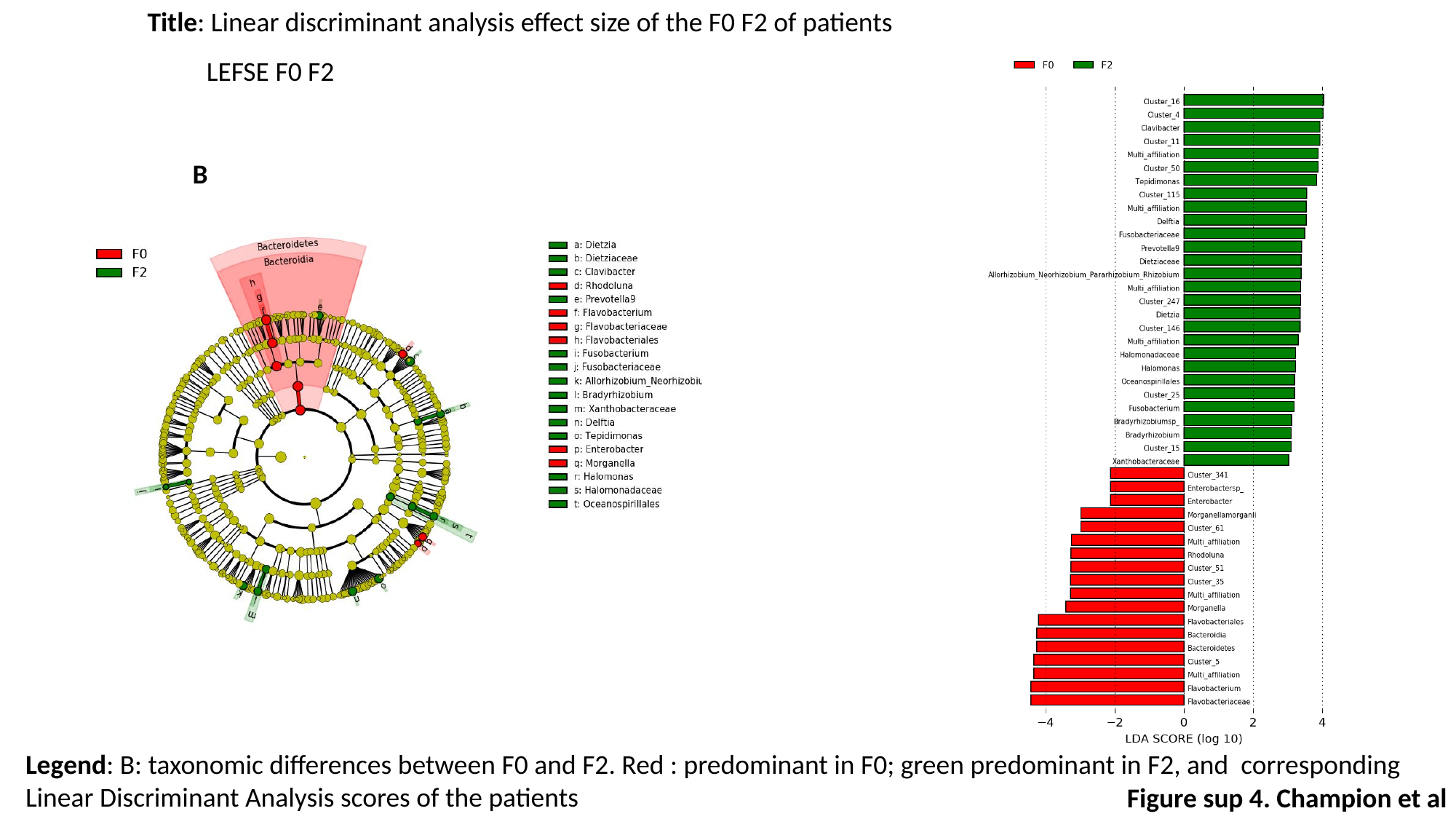

Title: Linear discriminant analysis effect size of the F0 F2 of patients
LEFSE F0 F2
B
Legend: B: taxonomic differences between F0 and F2. Red : predominant in F0; green predominant in F2, and corresponding Linear Discriminant Analysis scores of the patients
Figure sup 4. Champion et al
